# Sexual Dimorphism as a Potential Driver of Trophic Specialisation in a Sedentary Synanthropic Bat Species

**DOI:** 10.1002/ece3.74202

**Published:** 2026-08-16

**Authors:** Maryna Yerofieieva, Olena Holovchenko, Dina K. N. Dechmann, Anton Vlaschenko

**Affiliations:** ^1^ Ukrainian Bat Rehabilitation Center NGO ‘Ukrainian Independent Ecology Institute’ Kharkiv Ukraine; ^2^ Department of Migration Max Planck Institute of Animal Behavior Radolfzell Germany; ^3^ Department of Biology University of Konstanz Konstanz Germany; ^4^ Educational and Research Bat Biology Laboratory, Institute of Natural Sciences H.S. Skovoroda Kharkiv National Pedagogical University Kharkiv Ukraine; ^5^ Leibniz Institute for Zoo and Wildlife Research Berlin Germany; ^6^ National Scientific Center ‘Institute of Experimental and Clinical Veterinary Medicine’ Kharkiv Ukraine

**Keywords:** *Cnephaeus* (*Eptesicus*) *serotinus*, craniometry, intraspecific niche partitioning, morphometrics, urban ecology, Vespertilionidae, wing morphology

## Abstract

Intraspecific differences between the sexes can be as large as those between species, and niche partitioning between sexes may reduce competition for resources during energetically demanding periods. In bats, females are typically larger than males—a pattern attributed to greater reproductive investment (Big Mother hypothesis)—yet the functional implications of this dimorphism for foraging ecology remain poorly understood. We tested the hypothesis that sexual size dimorphism in cranial, wing and forearm morphology provides a functional basis for sex‐specific foraging ecology in *Cnephaeus* (*Eptesicus*) *serotinus*, a European sedentary synanthropic species with pronounced spatial segregation between sexes during the breeding season. Using craniometric measurements (*n* = 137), wing morphometrics (*n* = 22) and forearm length from long‐term monitoring (*n* = 1385) collected in northeastern Ukraine, we found consistent female‐biased dimorphism across all traits. Females were significantly larger in forearm length (+3.2%; Cohen's *d* = 1.07), with this pattern stable across 12 years of monitoring and consistent between age classes. Wing surface areas showed the greatest dimorphism (14%–15%; *d* = 1.6–2.0), while wing shape indices did not differ, indicating isometric size scaling consistent with compensation for pregnancy‐related mass increases. Mandibular dimensions related to bite force showed substantial dimorphism (2%–3%; *d* = 0.8–0.9), suggesting enhanced prey handling capacity in females. These findings are consistent with recent molecular dietary studies documenting sex‐specific prey consumption in other bat species. Our results provide morphological support for the hypothesis that sexual dimorphism enables both flight performance during pregnancy and intraspecific trophic niche partitioning, potentially reducing intersexual competition during reproduction.

## Graphical abstract

Female common serotine bats (*Cnephaeus serotinus*) are consistently larger than males in forearm length, wing area and mandibular dimensions related to bite force. Wing dimorphism reflects isometric size scaling rather than shape divergence, suggesting compensation for pregnancy‐related mass increases. Mandibular dimorphism indicates enhanced prey handling capacity in females, providing morphological support for intraspecific trophic niche partitioning.

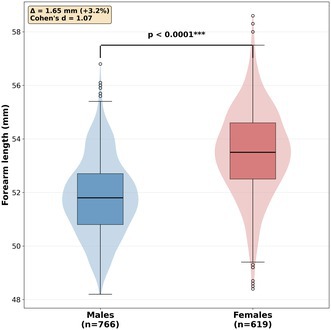

## 1 Introduction

Sexual dimorphism, a phenomenon marked by anatomical differences between males and females, has been a subject of research since the time of Charles Darwin (Darwin 1981), who proposed the theory of sexual selection to explain the evolution of these differences. Over time, various theories have emerged to examine the mechanisms and ecological significance of sexual dimorphism, and it is now widely accepted that these differences often represent a compromise between sexual and natural selection (Andersson 1994). Sexual dimorphism manifests in multiple forms, including body size, colouration, ornamentation and skeletal structure, and is observed across a broad range of taxa (Fairbairn et al. 2007).

Sexual size dimorphism (SSD) describes body size differences between sexes shaped by diverse selective pressures (Ralls 1976; Clutton‐Brock 1989). In most mammalian taxa, males are larger than females, primarily due to sexual selection favouring increased competitive ability (Weckerly 1998). However, this pattern is reversed in several groups, including many bat families, where females typically exceed males in body size (Myers 1978; Williams and Findley 1979). Several hypotheses have been proposed to explain this female‐biased SSD in bats, including the ‘Big Mother’ hypothesis, which posits that larger females have enhanced reproductive success through improved fecundity, thermoregulation and capacity to carry developing young during flight (Ralls 1976; Myers 1978). Understanding how these differences are linked to ecological adaptations—such as foraging strategies, resource partitioning and niche specialisation—remains a central area of research.

Bats represent a unique model system for studying morphological adaptations because they face distinctive constraints imposed by powered flight combined with viviparity (Norberg and Rayner 1987). Female bats must maintain flight capability while carrying foetuses that may constitute 25%–38% of maternal body mass in some species (Kurta and Kunz 1987). This challenge has led to predictions that females should possess morphological adaptations in wing structure to compensate for increased wing loading during pregnancy and lactation (Camargo and Oliveira 2012; Stevens et al. 2013; Stevens et al. 2016). Indeed, studies across various bat families have documented female‐biased dimorphism in forearm length, wing area and body mass, with the Vespertilionidae showing particularly consistent patterns of larger females (Myers 1978; Levin et al. 2013; Stevens and Platt 2015; O'Mara et al. 2016; Crampton et al. 2024). Wing morphology shapes flight performance and foraging behaviour, with wing loading and aspect ratio influencing manoeuvrability, flight speed and access to different foraging microhabitats (Norberg and Rayner 1987; Dietz et al. 2009). If wing dimorphism primarily operates through size scaling—with females possessing proportionally larger wings of the same shape—this would suggest compensation for pregnancy‐related mass increases rather than divergent flight strategies between sexes.

Beyond wing morphology, cranial structure—particularly mandibular morphology—is closely tied to trophic ecology and prey handling capacity in bats (Freeman 1981; Santana et al. 2010). Cranial traits, including coronoid height, condyle position and masseteric fossa dimensions, determine the mechanical advantage of jaw musculature, with larger values generally associated with greater bite force and ability to process harder or larger prey items (Aguirre et al. 2003; Ghazali and Dzeverin 2013; Ramírez‐Fráncel et al. 2022). Sexual dimorphism in craniometric characters has been documented in several bat species, with the magnitude and pattern of dimorphism varying among populations and subspecies (Dzeverin 1995a, 1995b; Crampton et al. 2024). However, such dimorphism is not universal among vespertilionids (Strelkov et al. 2002; Crampton et al. 2024), suggesting that its expression may depend on species‐specific ecological conditions or life‐history strategies. If sexes differ systematically in mandibular traits related to bite force, such differences could provide the functional basis for divergent prey selection and dietary specialisation, thereby facilitating intraspecific niche partitioning and reducing competition for limited resources between the sexes (Slatkin 1984). While interspecific competition in bats has been extensively studied—with niche partitioning identified in approximately 69% of investigated cases (Salinas‐Ramos et al. 2020)—intraspecific competition between sexes remains comparatively understudied.

Recent advances in molecular dietary analysis have begun to reveal sex‐specific differences in prey consumption that traditional morphological methods could not detect. Using DNA metabarcoding, Mata et al. (2016) demonstrated that female *Tadarida teniotis* (Molossidae) consumed larger moths than males, representing the first documented case of sex‐related dietary variation in bats. Similarly, Haarsma et al. (2023) found that diet composition in *Myotis dasycneme* differed significantly between sexes, with pregnant females selecting prey of different size classes compared to males. These findings suggest that morphological differences between sexes may translate into measurable dietary divergence, potentially reducing intersexual competition during energetically demanding reproductive periods.

Sexual segregation of males and females during the breeding season—involving both differential habitat use and seasonal shifts in sex composition—is a well‐documented phenomenon in many bat species of the Northern Hemisphere (Russo 2002; Senior et al. 2005; Estók 2007; Ibáñez et al. 2009; van Toor et al. 2011; Lintott et al. 2014; Vlaschenko et al. 2023). During gestation and lactation, females typically form maternity colonies and select more productive habitats such as wetlands, woodlands, valleys and river floodplains (Russo 2002; McGuire and Boyle 2013). In contrast, males are often more abundant in less productive environments, including upland areas, island forests and urban landscapes, where they tend to remain closer to overwintering sites or occupy solitary roosts (Nardone et al. 2015; Vlaschenko et al. 2023). This sexual segregation is widely interpreted as a consequence of the significantly higher energetic demands faced by reproductive females, which drive habitat selection toward areas that maximise foraging efficiency and support offspring development (Russo 2002; McGuire and Boyle 2013).

The common serotine, formerly *Eptesicus serotinus* (Schreber, 1774), currently *Cnephaeus serotinus* (Schreber, 1774) (Cláudio et al. 2023), represents an excellent model for investigating morphological dimorphism and its ecological implications. As a sedentary species that does not undertake long‐distance migrations (Hutterer et al. 2005; Steffens et al. 2004; Dietz et al. 2009), populations experience year‐round resource competition without the confounding effects of differential migratory behaviour between sexes. The species is highly synanthropic and well‐adapted to urban environments (Russo and Ancillotto 2015; Godlevska and Rebrov 2018; Martinoli et al. 2020), roosting predominantly in buildings across much of its European range (Martinoli et al. 2020). Long‐term studies in Kharkiv, Ukraine, have documented pronounced spatial segregation between sexes during the breeding season, with females largely absent from the urban core in spring and summer while males remain resident year‐round (Kravchenko et al. 2017; Vlaschenko et al. 2023). This spatial separation, combined with the species' documented dietary flexibility (Tiede et al. 2020), suggests conditions that are conducive to sex‐specific foraging strategies, which may be underpinned by morphological differentiation.

In this study, we test the hypothesis that sexual size dimorphism in *C. serotinus* provides a morphological basis for intraspecific trophic niche partitioning. We combine three morphological datasets—skull craniometry, wing morphometrics and forearm measurements—to comprehensively characterise dimorphism across morphological systems with distinct functional implications and test three non‐mutually exclusive hypotheses. First (H1), the ‘Big Mother’ hypothesis applies to *C. serotinus* , predicting that females should exceed males in body dimensions, particularly in traits linked to flight performance. Second (H2), sexual dimorphism facilitates intersexual trophic niche partitioning, predicting that mandibular dimensions associated with bite‐force generation should differ between sexes. Third (H3), wing dimorphism primarily compensates for pregnancy‐related mass increases rather than reflecting divergent flight strategies, predicting that intersexual differences should be more pronounced in wing area than in shape‐related indices. By linking morphological patterns to testable predictions, this study aims to clarify how intraspecific resource partitioning and reproductive selection jointly shape morphology in this widespread synanthropic bat.

## 2 Materials and Methods

All specimens originated from Kharkiv city and surrounding areas (northeastern Ukraine, approximately 50° N, 36° E). Specimens used for craniometric and wing analyses are housed in the Bat Carcasses Storing Collection of the UBRC; forearm length data were obtained from the UBRC long‐term monitoring database (Prylutska et al. 2023). We assembled three independent morphometric datasets: cranial morphometrics of skull and mandibular dimensions, wing morphometrics and forearm length. Every individual admitted to the UBRC undergoes a standardised examination upon intake, which includes age determination (Kravchenko et al. 2017; Prylutska et al. 2021). Age was assessed in all live and freshly dead bats; only decomposed or mummified carcasses, in which soft tissue degradation precludes reliable evaluation, were excluded from age assignment and were recorded as unknown age (un) in the database. Adults (ad) were defined as individuals that had survived at least one winter, corresponding to an age of approximately ten months or older (born in June, reaching this threshold by the following April). Subadults (sad) were defined as young‐of‐the‐year that had completed postnatal development and were capable of independent flight (typically from July onward, approximately one month after birth). Non‐volant juveniles were not represented in our sample.

Bats that died during rehabilitation, or were admitted dead, were preserved for the Bat Carcasses Storing Collection of the UBRC. Carcasses were frozen as soon as possible after death and stored in individually labelled bags at −15°C–18°C to −20°C, with each label recording date, locality, sex, age, body mass and forearm length at admission. In early 2022, following the onset of the full‐scale invasion and associated power outages that threatened freezer integrity (Mikołuszko 2022), a complete inventory of the collection was conducted. Each carcass was assessed and assigned to one of four condition categories: fresh (fully frozen with complete limb mobility upon thawing), medium (long‐term frozen specimens with partially desiccated wingtips), decayed (showing signs of soft tissue decomposition) and dry (desiccated or mummified). After inspection, all specimens were refrozen at −20°C.

For wing morphometrics, only specimens classified as fresh were used (Bininda‐Emonds and Russell 1994). Photography was conducted in May–June 2022. Carcasses were thawed in batches of 5–10 individuals at room temperature for approximately 1.5 h until full limb mobility was restored (see Section 3.2 below for details). After photography, specimens were refrozen at −20°C.

For each dataset, we assessed the potential confounding effect of age on sexual dimorphism. We performed a two‐way analysis of variance (ANOVA) on the subset of specimens with confirmed age, testing for the main effects of sex and age (adult vs. subadult) as well as their interaction (Sex × Age) on each morphometric parameter. The partial eta‐squared (*η* ^2^) was calculated as a measure of effect size for each factor. A non‐significant Sex × Age interaction (*p* > 0.05) was interpreted as evidence that the magnitude of sexual dimorphism is consistent across age classes, whereas a significant interaction would indicate that the pattern of dimorphism varies between adults and subadults. The number of specimens with confirmed age available for two‐way ANOVA was *n* = 97 for craniometric data (39 adults, 58 subadults), *n* = 22 for wing morphometrics (13 adults, 9 subadults) and *n* = 1323 for forearm length (757 adults, 566 subadults). The specimens represent the same population in which spatial segregation between sexes during the breeding season has been previously documented (Kravchenko et al. 2017; Vlaschenko et al. 2023).

### 2.1 Cranial Morphometrics Data

A total of 137 skulls of *C. serotinus* (70 females, 67 males) were examined (Table S1). During craniometric measurements, age classification was verified by assessing the degree of dental wear and cross‐referencing with the original database records (Kravchenko et al. 2017; Prylutska et al. 2021). When discrepancies were found or uncertainty remained, age was classified as unknown (un). Age was confirmed for 97 individuals: 39 adults (20 females, 19 males) and 58 subadults (31 females, 27 males).

**FIGURE 1:**
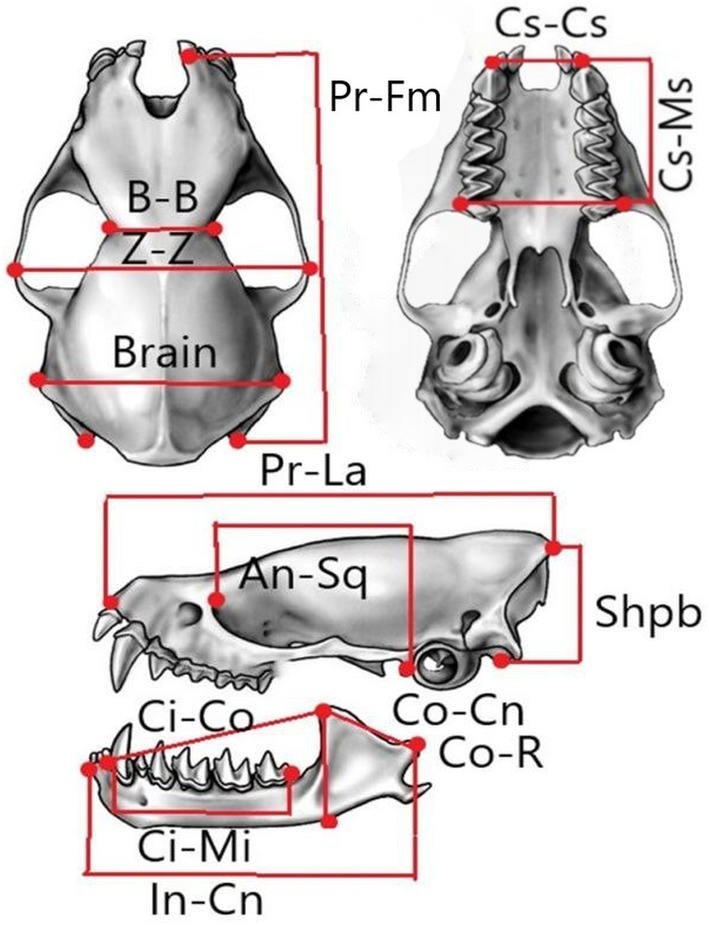
Description of craniometric measurements of *C. serotinus* : Skull: Pr‐Fm (condylobasal length), Pr‐La (greatest skull length), Z‐Z (zygomatic width), B‐B (minimum interorbital width), Brain (greatest width of the braincase), Shpb (skull height), An‐Sq (length of the zygomatic arch), Cs‐Mg (upper tooth row length), Mg‐Mg (width between third upper molars), Cs‐Cs (width between upper canines). Mandible: In‐Cn (total length of the mandible), Ci‐Co (distance from canine to coronoid process), Ci‐Mi (lower tooth row length) Co‐R (height of the coronoid process) Co‐Cn (distance between coronoid and condylar processes).

Fifteen craniometric measurements (Figure 1) were taken using a digital calliper (accuracy ±0.01 mm). Each measurement was repeated four to five times, and the mean value was used for analysis. To avoid bilateral redundancy, only right‐sided measurements were used for paired structures.

### 2.2 Cranial Morphometrics Statistical Analysis

All variables were tested for normality using the Shapiro–Wilk test. Two complementary analytical approaches were employed to assess sexual dimorphism in cranial morphology.

*Primary analysis*. Sex differences were assessed across the full sample (*n* = 137) using Welch's *t*‐test for normally distributed variables and the Mann–Whitney *U* test for non‐normally distributed data. To control for multiple comparisons, *p*‐values were adjusted using the Benjamini–Hochberg false discovery rate (FDR) correction. Statistical significance was set at *α* = 0.05.

*Secondary analysis*. To evaluate the potential confounding effect of age on sexual dimorphism, a two‐way analysis of variance (ANOVA) was performed on the subset of specimens with known age (*n* = 97). This analysis tested for the main effects of sex and age (adult vs. subadult), as well as their interaction (Sex × Age). The partial eta‐squared (*η* ^2^) was calculated as a measure of effect size for each factor.

To explore overall patterns of morphological variation and visualise sex‐based clustering, principal component analysis (PCA) was performed on standardised (*z*‐scored) craniometric data. PCA biplots were generated with 95% confidence ellipses for each sex to illustrate group separation.

Because young‐of‐the‐year captured in August are only 4–8 weeks old and may have incompletely ossified skulls, a sensitivity analysis was performed to assess whether their inclusion biases the dimorphism estimates. Between‐sex comparisons (Welch's *t*‐test, FDR‐corrected) were repeated on three subsets: the full sample (*n* = 137), the sample excluding August subadults and unknown‐age individuals (*n* = 112), and confirmed adults only (*n* = 39).

All statistical analyses were conducted in R version 4.3.1 (R Core Team 2023) using the base stats package for statistical tests (including two‐way ANOVA via the aov() function) and the ggplot2 package (Wickham 2016) for visualisation.

### 2.3 Wing Morphometrics Data and Parameters

Wing morphology was assessed postmortem in 22 individuals of *C. serotinus* (11 females, 11 males) selected from the UBRC Bat Carcasses Storing Collection (see above). The sample included 13 adults and 9 subadults. Prior to measurement, frozen carcasses were thawed at room temperature and placed ventral side up on a copy stand with both wings fully spread in a standardised anatomical position (Norberg and Rayner 1987), then photographed using a digital camera (Canon EOS 7D with a Canon EF‐S 18–55 mm zoom lens) mounted vertically above the specimen with a millimetre scale included in the frame (Figure S1a–c). Only the left wing was used for analysis.

**FIGURE 2:**
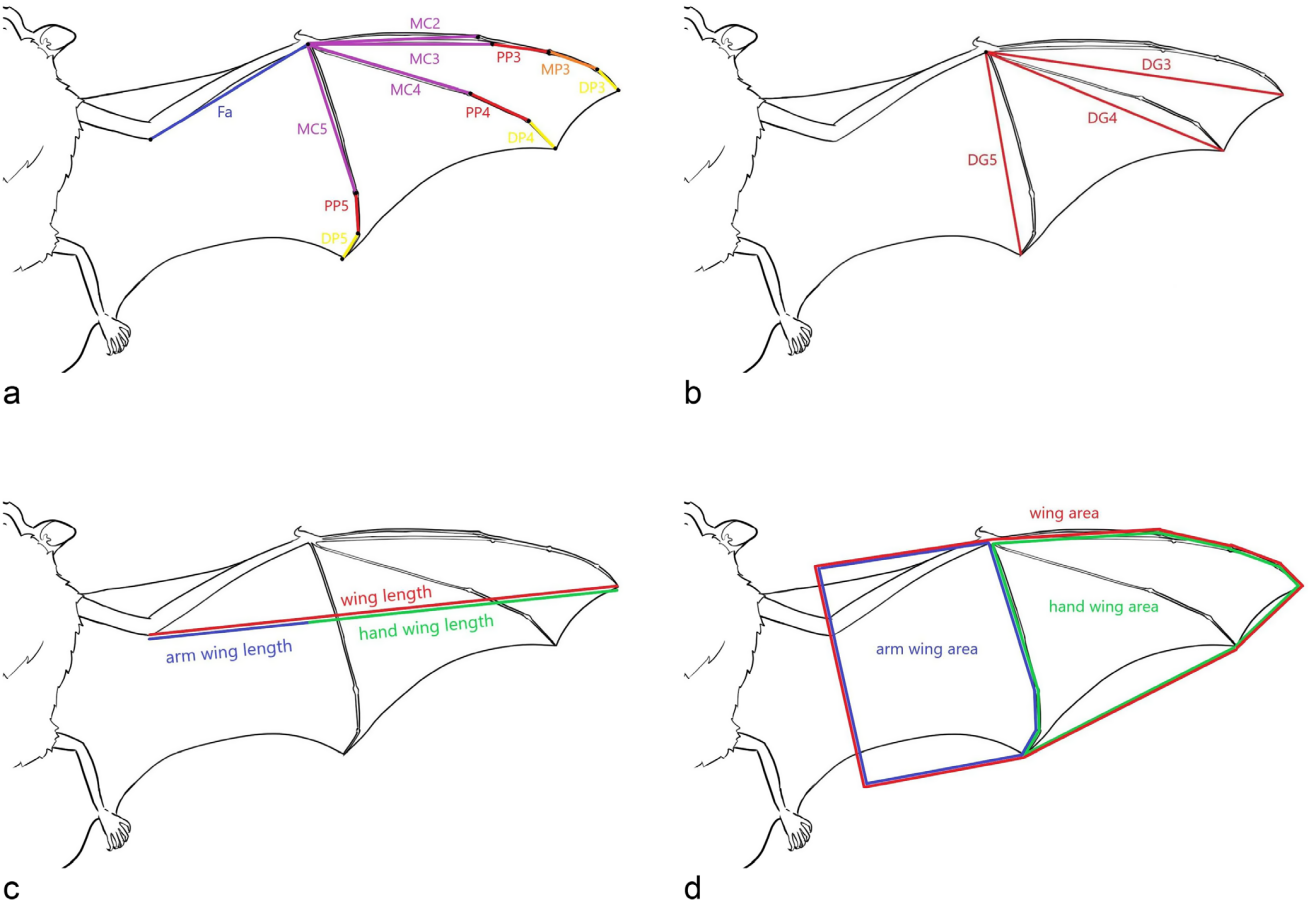
Description of measurements of wing parameters of *C. serotinus* : (a, b) Skeletal elements: Forearm length (Fa), metacarpals of digits 2–5 (MC2, MC3, MC4, MC5), proximal phalanges of digits 3–5 (PP3, PP4, PP5), medial phalanx of digit 3 (MP3), distal phalanges of digits 3–5 (DP3, DP4, DP5) and total digit lengths (DG3, DG4, DG5, calculated as the sum of metacarpal and phalanges for each digit); (c) Linear wing dimensions: Wing length (WL, distance between landmarks at the shoulder and wingtip), arm wing length (AL, linear distance from the elbow to the base of digit 5, measured perpendicular to the longitudinal axis of the body (c)) and hand wing length (HL, length of the dactylopatagium from digit 5 to the wingtip); (d) Surface areas: Total wing area (WA), arm wing area (AA, area of the proximal portion of the wing, corresponding to the plagiopatagium as shown in (d)) and hand wing area (HA, surface of the dactylopatagium from digit 5 to the wingtip). The boundary between the arm wing and the hand wing was defined along digit 5.

A total of 21 morphometric parameters (Figure 2) were measured, comprising skeletal elements, linear wing dimensions and surface areas (Table S2). All measurements were performed on digitised photographs using ImageJ software (Schneider et al. 2012). To assess measurement repeatability, a subset of 10 specimens was measured five times by the same observer using identical landmarks and scale calibration. Repeatability was evaluated using one‐way analysis of variance (ANOVA) for each parameter. No statistically significant differences among repeated measurements were detected for any variable (all *p* > 0.05), confirming high measurement consistency. Three wing shape indices were calculated from the primary measurements: aspect ratio (AR = WL^2^/WA), tip length ratio (TL = HL/AL) and tip area ratio (TA = HA/AA), following Norberg and Rayner (1987). These indices describe overall wing shape independently of absolute size and were used to test whether sexual dimorphism operates through isometric size scaling or involves shape divergence (H3).

### 2.4 Wing Morphometrics Statistical Analysis

All variables were tested for normality using the Shapiro–Wilk test. Sex differences were assessed using Welch's *t*‐test for normally distributed variables and the Mann–Whitney *U* test for non‐normally distributed data. Statistical significance was set at *α* = 0.05.

To evaluate the potential confounding effect of age on sexual dimorphism, two‐way analysis of variance (ANOVA) was performed for each parameter, testing for the main effects of sex and age (adult vs. subadult) as well as their interaction (Sex × Age). The partial eta‐squared (*η* ^2^) was calculated as a measure of effect size.

All statistical analyses were conducted in R version 4.3.1 (R Core Team 2023), and visualisation was performed using ggplot2 (Wickham 2016).

### 2.5 Forearm Length Data

Forearm length data were obtained from the UBRC long‐term monitoring database comprising 1385 individuals of *C. serotinus* (619 females, 766 males), adults (ad, *n* = 757), young‐of‐the‐year (sad, *n* = 566) and unknown age (un, *n* = 57), collected in Kharkiv city between 2013 and 2024. Forearm length (Ra) was measured from the elbow to the wrist using a digital calliper (accuracy ±0.1 mm) following standard chiropterological protocols (Prylutska et al. 2021).

### 2.6 Forearm Length Statistical Analysis

All variables were tested for normality using D'Agostino–Pearson test. Due to slight deviations from normality in males, both parametric (Welch's *t*‐test) and non‐parametric (Mann–Whitney *U* test) comparisons were performed. Effect size was quantified using Cohen's *d*, with values of 0.2, 0.5 and 0.8 interpreted as small, medium and large effects, respectively (Cohen 1988).

To evaluate the effects of sex and age on forearm length, two‐way analysis of variance (ANOVA) was performed on specimens with known age (*n* = 1323), testing for main effects of sex and age (adult vs. subadult) and their interaction (Sex × Age). Partial eta‐squared (*η* ^2^) was calculated as a measure of effect size for each factor.

## 3 Results

### 3.1 Cranial Morphometrics

Analysis of 137 skulls (70 females, 67 males) revealed significant female‐biased sexual dimorphism in *C. serotinus* . Of the 15 craniometric parameters examined (Figure 1), 12 (80%) showed statistically significant sex differences after FDR correction (Table 1). In all cases where significant dimorphism was detected, females were larger than males.

**TABLE 1:** Cranial measurements in *C. serotinus* (mean ± SD).

Parameter	Males (mm)	Females (mm)	Δ%	*d*	*p*
Mandible length (In‐Cn)	15.31 ± 0.34	15.64 ± 0.38	+2.2	0.92	**0.00001**
Coronoid‐condylar distance (Co‐Cn)	4.98 ± 0.17	5.15 ± 0.21	+3.4	0.89	**0.00001**
Coronoid height (Co‐R)	5.86 ± 0.18	6.05 ± 0.28	+3.4	0.86	**0.00001**
Lower tooth row length (Ci‐Mi)	8.44 ± 0.20	8.60 ± 0.21	+1.9	0.78	**0.00001**
Toothrow‐mastoid (Cs‐Mg)	7.48 ± 0.16	7.60 ± 0.20	+1.5	0.64	**0.0004**
Auditory‐squamosal (An‐Sq)	10.16 ± 0.26	10.33 ± 0.29	+1.6	0.61	**0.0005**
Mastoid width (Mg‐Mg)	8.52 ± 0.21	8.65 ± 0.25	+1.5	0.58	**0.0012**
Total skull length (Pr‐Fm)	19.84 ± 0.40	20.09 ± 0.46	+1.3	0.58	**0.0025**
Zygomatic width (Z‐Z)	14.04 ± 0.52	14.25 ± 0.47	+1.5	0.41	**0.0267**
Canine width (Cs‐Cs)	6.41 ± 0.21	6.53 ± 0.19	+1.8	0.59	**0.0011**
Canine‐coronoid distance (Ci‐Co)	11.35 ± 0.32	11.57 ± 0.47	+2.0	0.56	**0.0055**
Skull length (Pr‐La)	20.65 ± 0.43	20.86 ± 0.51	+1.0	0.45	**0.0310**
Sphenoid breadth (Shpb)	6.69 ± 0.25	6.78 ± 0.21	+1.3	0.37	0.0582
Interorbital width (B‐B)	4.43 ± 0.19	4.40 ± 0.14	−0.8	−0.22	0.2160
Braincase breadth (Brain)	9.49 ± 0.22	9.55 ± 0.25	+0.6	0.24	0.1753

*Note:* Δ% = percent difference (F − M)/M; *d* = Cohen's *d*. Bold *p*‐values indicate statistical significance after FDR correction (*p* < 0.05).

The most pronounced dimorphism was observed in mandibular measurements. Coronoid height (Co‐R) and coronoid‐condylar length (Co‐Cn) showed the greatest differences, with females averaging 3.4% larger than males in both parameters (*p* < 0.001 Cohen's *d* = 0.85 and 0.91, respectively). Mandible length (In‐Cn) was 2.2% greater in females (*p* < 0.001, *d* = 0.94). Other mandibular parameters, including lower tooth row length (Ci‐Mi, +1.9%) and canine‐coronoid distance (Ci‐Co, +2.0%), also showed significant female‐biased dimorphism (all *p* < 0.01) (Table 1).

Cranial dimensions exhibited more moderate dimorphism (Table 1). Zygomatic width (Z‐Z) was 1.5% greater in females (*p* = 0.002), while mastoid width (Mg‐Mg), canine width (Cs‐Cs) and maxillary toothrow‐mastoid distance (Cs‐Mg) were 1.5%–1.8% larger (all *p* < 0.01). The skull length (Pr‐La) and total skull length (Pr‐Fm) showed 1.0% and 1.3% greater values in females, respectively (*p* < 0.05). Three parameters showed no significant sexual dimorphism: braincase breadth (Brain, *p* = 0.188), interorbital width (B‐B, *p* = 0.253) and sphenoid breadth (Shpb, *p* = 0.067) (Table 1).

Two‐way ANOVA performed on specimens with known age (*n* = 97) confirmed that sex had a significant effect on 10 of 15 parameters, while age (adult vs. subadult) significantly affected only two parameters (Cs‐Cs and Pr‐La) (Figure 4). Importantly, the Sex × Age interaction was not significant for any parameter, indicating that the pattern of sexual dimorphism remains consistent across age classes. The partial eta‐squared values for sex ranged from 0.005 (Brain) to 0.188 (Co‐Cn), with mandibular parameters showing the largest effect sizes (*η* ^2^ = 0.10–0.19) (Table S3).

**FIGURE 3:**
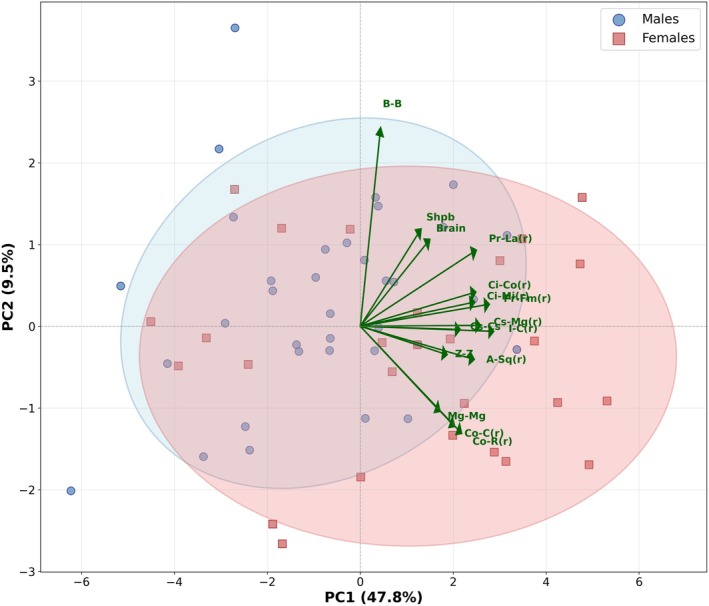
Principal component analysis (PCA) biplot of 15 craniometric measurements in *C. serotinus* (*n* = 137). Males (blue circles) and females (red squares) are shown with 95% confidence ellipses. Arrows represent variable loadings. PC1 (47.8%) separates sexes, with females at positive values; highest loadings include mandibular (In‐Cn Co‐Cn Co‐R) and skull length measurements (Pr‐Fm). PC2 (9.5%) reflects braincase shape variation (B‐B, Shpb) independent of sex.

**FIGURE 4:**
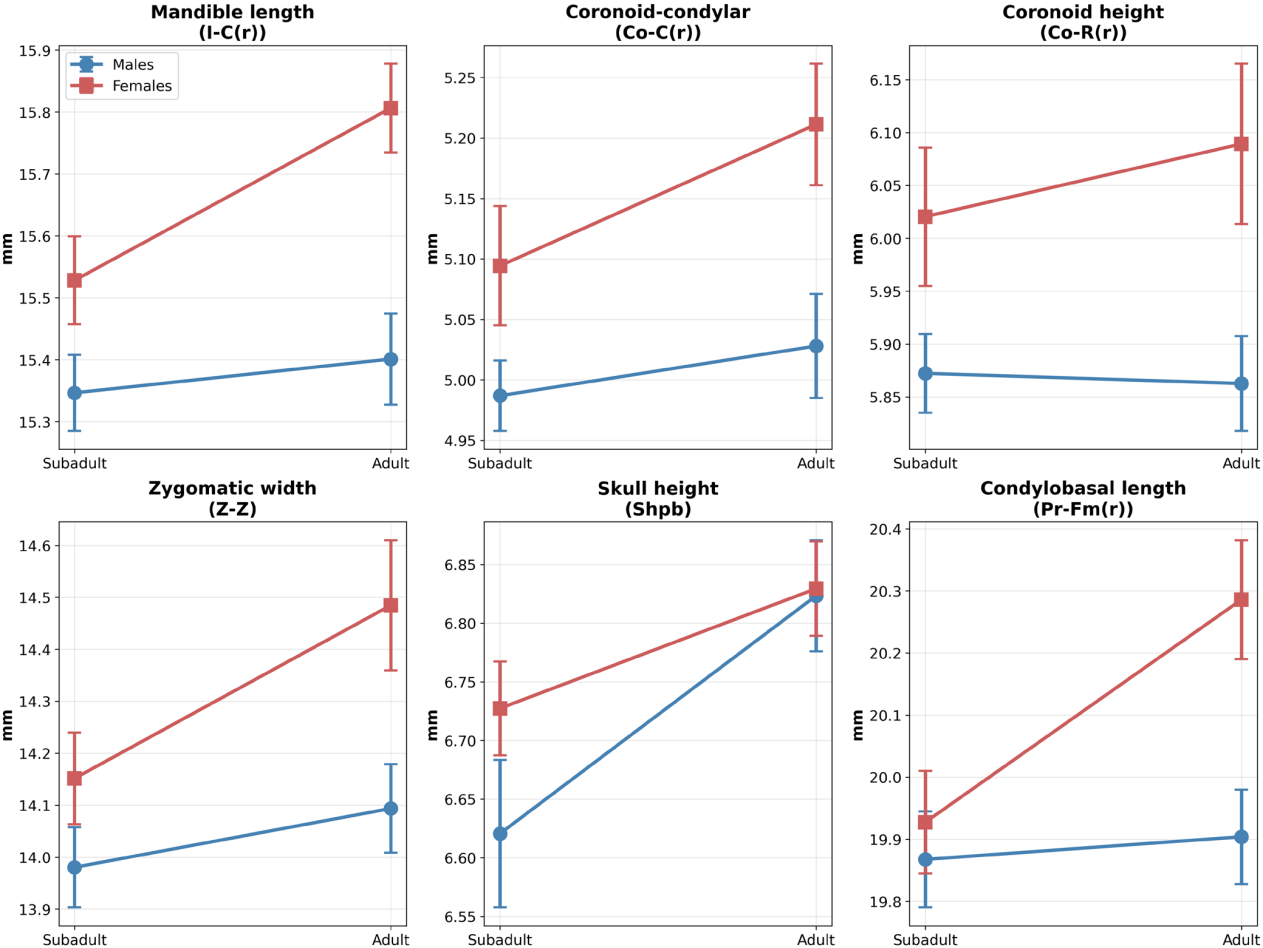
Effect of sex and age on selected cranial measurements in *C. serotinus* (mean ± SE). Females (red) are consistently larger than males (blue) across all parameters. Parallel lines indicate no significant Sex × Age interaction (two‐way ANOVA, *p* > 0.05 for all). Both sexes show a slight increase from subadult to adult in most parameters.

Principal component analysis of all 15 craniometric parameters showed that PC1 explained 47.8% of the total variance, while PC2 accounted for 9.5% (Figure 3). The PCA biplot revealed moderate separation between sexes along PC1, with considerable overlap between male and female clusters. Variables with the highest loadings on PC1 included mandibular parameters (In‐Cn Co‐Cn Co‐R) and skull length measurements (Pr‐La, Pr‐Fm), consistent with the univariate results showing these as the most dimorphic traits (Figure 4).

The pattern of sexual dimorphism was highly robust to the exclusion of potentially immature August specimens (Table S5). The number of significant variables decreased from 13/16 (full sample) to 11/16 (excluding August young) and 7/16 (adults only), with the latter reduction largely reflecting reduced statistical power (*n* = 39) (Table S6). Crucially, the direction of dimorphism was preserved for every variable across all three subsets, and effect sizes changed only marginally (mean |Δ*d*| = 0.05). For the key mandibular dimensions (In‐Cn, Ci‐Mi, Ci‐Co Co‐Cn Co‐R), forearm length, and zygomatic arch length, dimorphism remained significant (FDR‐adjusted *p* < 0.01) in all three subsets.

### 3.2 Wing Morphometrics

Wing measurements from 22 individuals (11 females, 11 males) revealed pronounced female‐biased sexual dimorphism in wing size (Table 2). Of the 21 wing parameters measured, 13 (62%) showed significant sex differences (*p* < 0.05).

**TABLE 2:** Wing measurements in *C. serotinus* (*n* = 11 per sex; mean ± SD).

Parameter	Males	Females	Δ%	*d*	*p*
Wing area, cm^2^ (WA)	62.33 ± 3.70	71.15 ± 4.86	+14.2	2.04	**< 0.0001**
Hand wing area, cm^2^ (HA)	31.49 ± 2.33	36.18 ± 2.65	+14.9	1.88	**0.0003**
Arm wing area, cm^2^ (AA)	30.96 ± 2.51	35.02 ± 2.55	+13.1	1.60	**0.0012**
Wing length, cm (WL)	13.19 ± 0.34	13.79 ± 0.50	+4.5	1.40	**0.0043**
Arm wing length, cm (AL)	4.47 ± 0.19	4.82 ± 0.39	+8.0	1.17	**0.0154**
Hand wing length, cm (HL)	8.73 ± 0.20	8.96 ± 0.31	+2.6	0.86	0.0597
Forearm, cm (Fa)	5.00 ± 0.15	5.20 ± 0.15	+4.0	1.32	**0.0057**
Metacarpal 2, cm (MC2)	4.59 ± 0.16	4.80 ± 0.37	+4.6	0.74	0.1068
Metacarpal 3, cm (MC3)	4.89 ± 0.13	5.08 ± 0.15	+4.0	1.38	**0.0043**
Metacarpal 4, cm (MC4)	4.76 ± 0.15	4.96 ± 0.18	+4.1	1.19	**0.0117**
Metacarpal 5, cm (MC5)	4.43 ± 0.14	4.67 ± 0.19	+5.3	1.40	**0.0040**
Digit 3, cm (DG3)	8.81 ± 0.25	9.12 ± 0.31	+3.5	1.10	**0.0183**
Digit 4, cm (DG4)	7.46 ± 0.19	7.51 ± 1.01	+0.7	0.07	0.8772
Digit 5, cm (DG5)	6.17 ± 0.24	6.53 ± 0.24	+5.7	1.47	**0.0025**
Proximal phalanx 3, cm (PP3)	1.81 ± 0.09	1.89 ± 0.09	+4.4	0.90	**0.0468**
Medial phalanx 3, cm (MP3)	1.52 ± 0.17	1.55 ± 0.09	+1.9	0.21	0.6282
Distal phalanx 3, cm (DP3)	1.01 ± 0.17	1.14 ± 0.17	+13.0	0.76	0.0892
Proximal phalanx 4, cm (PP4)	1.62 ± 0.12	1.71 ± 0.10	+5.6	0.82	0.0703
Distal phalanx 4, cm (DP4)	1.27 ± 0.06	1.33 ± 0.14	+5.3	0.61	0.1761
Proximal phalanx 5, cm (PP5)	1.22 ± 0.08	1.28 ± 0.14	+4.9	0.51	0.2471
Distal phalanx 5, cm (DP5)	0.91 ± 0.11	0.99 ± 0.21	+8.8	0.47	0.2869
Aspect ratio (AR)	2.80 ± 0.10	2.68 ± 0.13	−4.1	−1.00	0.0570
Tip length ratio (TL)	1.96 ± 0.10	1.87 ± 0.16	−4.6	−0.68	0.1120
Tip area ratio (TA)	1.02 ± 0.09	1.04 ± 0.08	+1.4	0.14	0.7620

*Note:* Δ% = percent difference (F − M)/M; *d* = Cohen's *d*. Bold *p*‐values indicate statistical significance after FDR correction (*p* < 0.05).

**FIGURE 5:**
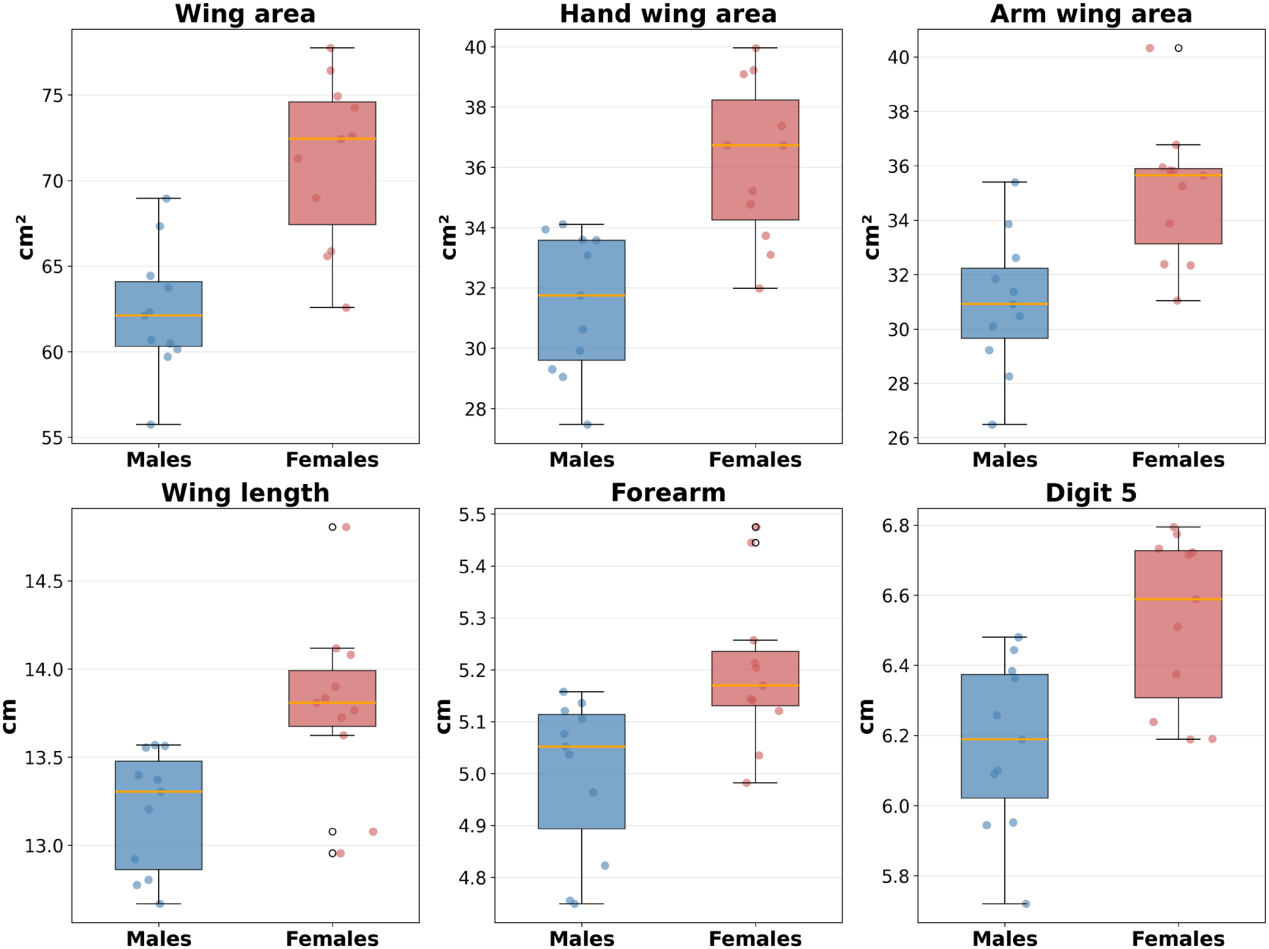
Comparison of some wing measurements between sexes in *C. serotinus* (*n* = 11 per sex). Wing areas (top row) show the greatest dimorphism (13%–15%, *p* < 0.01). Linear measurements (bottom row) show moderate dimorphism (4%–6%). Individual data points are shown with jitter.

The most striking differences were observed in wing surface areas. Total wing area (WA) was 14.2% larger in females than in males (71.15 ± 4.86 cm^2^ vs. 62.33 ± 3.70 cm^2^; *p* < 0.001, Cohen's *d* = 2.04). Similarly, hand wing area (HA) was 14.9% larger in females (*p* < 0.001, *d* = 1.88), and arm wing area (AA) was 13.1% larger (*p* = 0.001, *d* = 1.60). Linear wing dimensions showed more moderate dimorphism. Wing length (WL) was 4.5% greater in females (13.79 ± 0.50 cm vs. 13.19 ± 0.34 cm; *p* = 0.004), while arm wing length (AL) showed 8.0% larger values in females (*p* = 0.015). Forearm length (Fa) measured from wing photographs was 4.0% larger in females (*p* = 0.006). Among skeletal elements, metacarpals MC3, MC4 and MC5 were 4.0%–5.3% larger in females (all *p* < 0.05), as was digit 5 (DG5, +5.7%, *p* = 0.003). Hand wing length (HL) showed a trend toward larger values in females (+2.6%) but did not reach statistical significance (*p* = 0.060) (Figure 5).

Notably, derived wing shape indices did not differ significantly between sexes. Aspect ratio (WL^2^/WA; *p* = 0.057), tip length ratio (HL/AL; *p* = 0.112) and tip area ratio (HA/AA; *p* = 0.762) were all similar between males and females. Two‐way ANOVA revealed that sex significantly affected 12 of 21 wing parameters (Table 2; Table S4), while age had a significant effect only on wing areas (WA, HA, AA). A significant Sex × Age interaction was detected for forearm length (Fa, *p* = 0.032), total wing area (WA, *p* = 0.043) and hand wing area (HA, *p* = 0.006), suggesting that the magnitude of sexual dimorphism in these parameters may vary somewhat between age classes.

**FIGURE 6:**
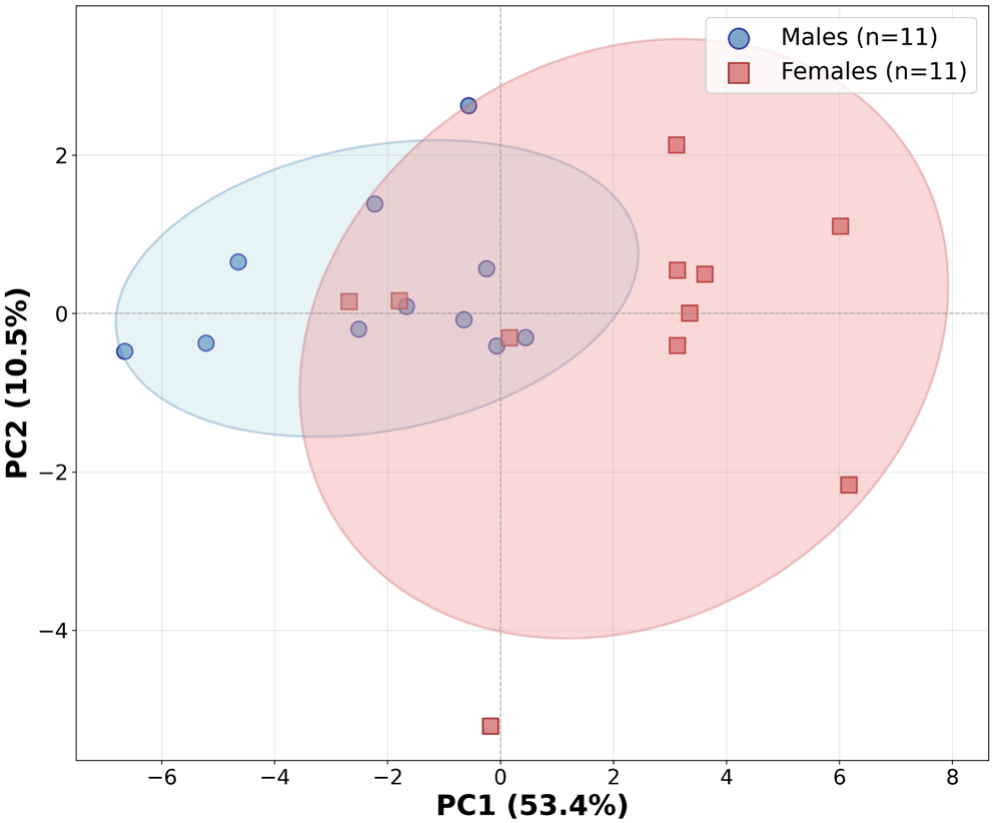
Principal Component Analysis (PCA) of 21 wing measurements in *C. serotinus* (*n* = 22). Males (blue circles) and females (red squares) with 95% confidence ellipses. PC1 (53.4%) separates sexes, with females at positive values. PC2 (10.5%) reflects individual variation independent of sex.

The results of PCA of wing measurements showed strong separation between sexes (Figure 6). PC1 explained 53.4% of total variance, with females clustering at positive PC1 values and males at negative values.

### 3.3 Forearm Length

**FIGURE 7:**
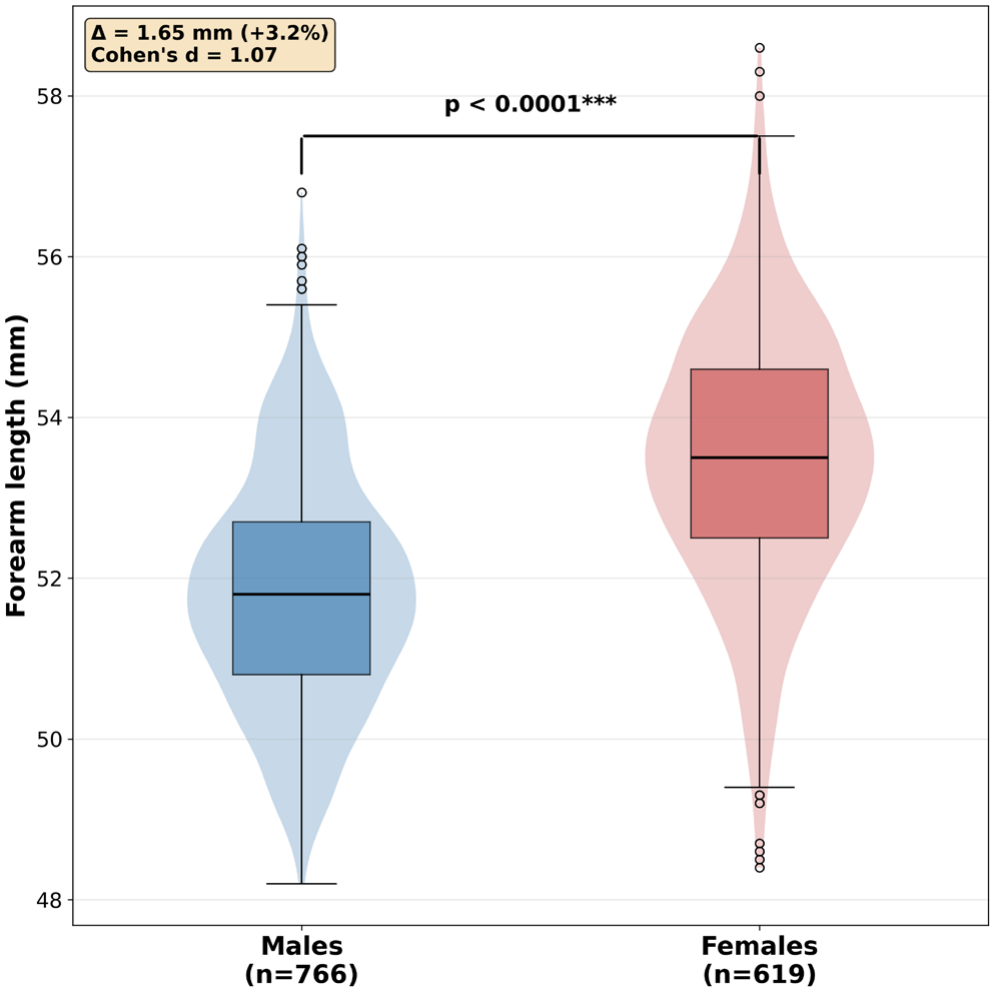
Sexual dimorphism in forearm length in *C. serotinus* (*n* = 1385). Violin plots show data distribution; box plots show median and interquartile range. Females are significantly larger than males (Δ = 1.65 mm, +3.2%; Cohen's *d* = 1.07; ***p < 0.0001).

Analysis of the large monitoring dataset (*n* = 1385; 619 females, 766 males) provided robust confirmation of sexual dimorphism in forearm length (Figure 7). Female forearm length averaged 53.47 ± 1.64 mm, significantly larger than males at 51.82 ± 1.45 mm (Welch's *t*‐test: *t* = 19.59, *p* < 0.0001; Mann–Whitney *U* = 369,780, *p* < 0.0001). The difference of 1.65 mm represents a 3.2% increase in females relative to males, with a large effect size (Cohen's *d* = 1.07).

Two‐way ANOVA on specimens with known age (*n* = 1323) revealed highly significant effects of both sex (*F* = 376.5, *p* < 0.0001, *η* ^2^ = 0.219) and age (*F* = 22.2, *p* < 0.0001, *η* ^2^ = 0.013). Adults of both sexes had slightly longer forearms than subadults (females: 53.70 vs. 53.14 mm; males: 51.89 vs. 51.77 mm). The Sex × Age interaction was not significant (*p* > 0.05), indicating that the magnitude of sexual dimorphism is consistent between adults and subadults.

Temporal analysis across 12 years of data collection (2013–2024) demonstrated remarkable stability of sexual dimorphism. In every year, female forearm length consistently exceeded male values by approximately 1.5–2.0 mm, with no significant temporal trend in the magnitude of dimorphism.

## 4 Discussion

Our study provides comprehensive morphometric evidence for pronounced female‐biased sexual size dimorphism in *C. serotinus* across three morphological systems: cranium, wing and forearm. All three hypotheses received support from our data. Females were significantly larger than males across most measured traits (H1). Mandibular measurements related to bite force generation showed substantial dimorphism, suggesting enhanced prey handling capacity in females (H2). Wing shape indices did not differ between sexes despite pronounced differences in wing area, indicating isometric size scaling rather than divergent flight strategies (H3). We propose that these morphological differences may provide the functional basis for spatial segregation between sexes during the breeding season in this sedentary synanthropic species, enabling males to remain in urban areas during summer while females relocate to more productive natural habitats.

### 4.1 Female‐Biased Sexual Size Dimorphism and the Big Mother Hypothesis

Analysis of the large monitoring dataset (*n* = 1385) provided robust confirmation of female‐biased sexual size dimorphism in *C. serotinus* , with females averaging 3.2% larger in forearm length than males (Cohen's *d* = 1.07). This dimorphism was stable across 12 years of monitoring (2013–2024) and consistent between age classes (no significant Sex × Age interaction), confirming that the observed pattern represents a robust biological feature of the species. Our findings contribute to the ongoing discussion of Rensch's rule in Chiroptera, which predicts that in clades with female‐biased SSD, the magnitude of dimorphism should decrease with increasing body size (Rensch 1950; Fairbairn 1997). The applicability of this rule to bats remains contentious. At the intraspecific level, some studies have found support (Wu et al. 2014; Storz et al. 2001), while interspecific analyses consistently fail to support the rule in Rhinolophidae (Wu et al. 2018), New World Myotis (Stevens and Platt 2015) and Neotropical bats (Castillo‐Figueroa 2024). Taxa with female‐biased dimorphism may systematically deviate from Rensch's rule (Webb and Freckleton 2007). *Cnephaeus serotinus*, as a relatively large vespertilionid (~19–28 g), exhibits pronounced female‐biased dimorphism (+3.2% in forearm length, +14%–15% in wing area), which does not conform to the prediction that dimorphism should diminish at larger body sizes. This deviation likely reflects the strong selective pressure imposed by the energetic demands of flight during pregnancy, as predicted by the ‘Big Mother’ hypothesis (Ralls 1976; Myers 1978; Williams and Findley 1979). Larger female body size may enhance reproductive success through improved thermoregulation during gestation and greater capacity to carry developing young during flight.

### 4.2 Wing Size Dimorphism

A notable finding of our study is the marked difference in effect sizes across morphological systems. Wing area dimorphism exhibited very large effect sizes (Cohen's *d* = 1.6–2.0), substantially exceeding those for cranial measurements (*d* = 0.4–0.9) and forearm length (*d* = 1.07). This pattern suggests that different selective pressures operate on different morphological systems (Stevens et al. 2013). The particularly strong dimorphism in wing area likely reflects the critical importance of flight performance during pregnancy—a period when females must carry substantial additional mass (up to 25%–38% of body weight in some species; Kurta and Kunz 1987) while maintaining sufficient manoeuvrability for aerial hawking. The particularly pronounced dimorphism in wing area (Cohen's *d* > 1.6) is consistent with the flight load hypothesis, a bat‐specific extension of the Big Mother hypothesis predicting that females should possess larger wings to compensate for increased wing loading during pregnancy (Camargo and Oliveira 2012; Stevens et al. 2013).

Critically, wing shape indices—including aspect ratio, tip length ratio and tip area ratio—did not differ significantly between sexes despite the pronounced differences in wing area. This indicates that dimorphism operates through isometric size scaling: females possess proportionally larger wings of the same shape rather than aerodynamically distinct wings. This finding is consistent with the prediction that wing dimorphism primarily compensates for increased mass during pregnancy rather than reflecting divergent flight strategies between sexes. A similar pattern was documented in *Nyctalus noctula* , where O'Mara et al. (2016) found sexual dimorphism in body size but not in wing shape, suggesting that isometric wing scaling may represent a widespread mechanism among vespertilionid bats for accommodating sex‐specific mass differences. Similarly, Cichocki et al. (2026) reported female‐biased wing dimorphism in *Myotis myotis* at the northern limit of its range, further supporting the Big Mother hypothesis across European vespertilionids.

### 4.3 Mandibular Dimorphism

Among cranial structures, mandibular measurements showed the strongest dimorphism, particularly coronoid height (Co‐R, +3.4%) and condyle‐coronoid distance (Co‐Cn, +3.4%) (Bogdanowicz et al. 1999; Nogueira et al. 2009). The prominence of lower jaw dimorphism over other cranial elements has clear functional significance. The mandible is the primary structure determining bite force generation: coronoid height affects the moment arm of the temporalis muscle, while condyle‐coronoid distance influences the mechanical advantage of jaw closure (Aguirre et al. 2003; Santana and Cheung 2016). By contrast, upper skull dimensions showed weaker dimorphism, suggesting that selection has acted more strongly on structures directly involved in prey capture and processing rather than on overall skull size. We note that this comparison is descriptive, based on the observed pattern of Cohen's *d* values (*d* = 0.78–0.92 for mandibular parameters vs. *d* = 0.24–0.64 for other cranial dimensions; Table 1), rather than on a formal statistical test of regional differences in dimorphism magnitude. This pattern is consistent with the demonstrated functional link between mandibular morphology and diet hardness in vespertilionid bats (Arbour et al. 2019; Ghazali and Dzeverin 2013; Ayala‐Berdon et al. 2023), and with niche partitioning hypotheses that predict differential prey selection between sexes based on handling capacity (Nogueira et al. 2009). Notably, mandibular dimorphism has previously been documented in *M. dasycneme* , with females possessing longer mandibles than males (Dzeverin 1995a)—the same species in which Haarsma et al. (2023) subsequently demonstrated sex‐specific dietary differences using molecular methods. This convergence of morphological and dietary evidence across independent studies provides indirect support for the hypothesis that mandibular dimorphism may underpin trophic niche partitioning between sexes.

Crampton et al. (2024) analysed mandibular morphology across four European bat species with differing foraging ecologies, including three vespertilionids— *M. daubentonii* (6–10 g, trawling), *P. austriacus* (7–14 g, gleaning) and *N. noctula* (21–40 g, aerial hawking)—and one rhinolophid, *Rhinolophus ferrumequinum* . Notably, sexual dimorphism in mandibular morphology was detected only in *R. ferrumequinum* , while none of the three vespertilionid species showed significant differences between sexes. The pronounced mandibular dimorphism we documented in *C. serotinus* (particularly in structures associated with bite force generation) represents a departure from the apparent lack of such dimorphism in other European vespertilionids. This contrast is especially striking when comparing *C. serotinus* with its ecologically similar congener *N. noctula* : both are large‐bodied aerial hawkers of comparable size, but *N. noctula* exhibits minimal sexual size dimorphism—females are only marginally larger (~1% in forearm length; O'Mara et al. 2016), with no significant differences in wing shape, mandibular morphology (Holovchenko et al. 2017; Crampton et al. 2024) or craniometric characters (Strelkov et al. 2002; Holovchenko et al. 2017).

### 4.4 Spatial Segregation of Different Sexes

The morphological dimorphism we documented takes on particular ecological significance in light of the pronounced spatial segregation between sexes observed in *C. serotinus* . Long‐term monitoring in the Ukrainian city of Kharkiv has demonstrated that both active mist‐netting surveys and opportunistic rescue records reveal a remarkably consistent pattern across multiple years and data sources (Kravchenko et al. 2017; Vlaschenko et al. 2023). From May onwards, captures and rescues of *C. serotinus* in the urban core consist almost exclusively of males (> 90%), indicating that females vacate built‐up areas in spring to establish maternity colonies elsewhere—presumably in more productive foraging habitats at the urban periphery or in surrounding rural areas. This male‐dominated pattern persists throughout the breeding season (May–July) until August, when adult females begin returning to the city together with their volant this‐year‐born pups, and the mating season starts (Fasel et al. 2023). By winter, the sex ratio approaches parity (55% females, 45% males) (Kravchenko et al. 2017; Vlaschenko et al. 2023), suggesting that urban areas serve as communal hibernation sites for both sexes. Comparable patterns of seasonal spatial segregation, with females forming maternity colonies outside dense settlements and males occurring singly or in small groups within built‐up areas, have been repeatedly reported across the species' range in Western and Central Europe (Catto et al. 1995; Robinson and Stebbings 1997; Dietz et al. 2007; Arthur and Lemaire 2009). This convergence suggests that the male‐dominated urban presence observed in Kharkiv reflects a general, species‐wide pattern for big cities rather than a local peculiarity.

Within this broader framework, sexual size dimorphism may underpin complementary ecological specialisation rather than simple competitive asymmetry between sexes. Females, characterised by larger wing areas and more robust mandibles, likely benefit from exploiting highly productive foraging habitats such as wetlands, floodplains and forest edges, which support higher insect biomass and larger prey during energetically demanding periods of pregnancy and lactation (Catto et al. 1996; Kervyn and Libois 2008; Martinoli et al. 2020). Conversely, the smaller body size and lower absolute energetic requirements of males may confer an advantage in less productive environments, including urban landscapes where insect assemblages are often dominated by small‐bodied taxa (Baagøe 1986; Robinson and Stebbings 1997; Chauvenet et al. 2014). From this perspective, the persistence of males in cities throughout most of the year may represent an adaptive strategy, allowing efficient exploitation of prey‐poor habitats that are suboptimal for reproductively active females. This spatial partitioning may reduce intersexual competition for food resources during pregnancy and lactation.

Importantly, current understanding of sex‐specific ecology in *C. serotinus* is strongly biased toward the contemporary period of extensive urbanisation. Mid‐20th century literature described *C. serotinus* as a montane species (Abelentsev et al. 1956), yet the spatial distribution of sexes in non‐urban habitats has not been described for this species. Nevertheless, elevational segregation of sexes, with males occurring at higher altitudes or in more climatically challenging environments, has been documented in other bat species (e.g., Russo 2002; Senior et al. 2005; Nardone et al. 2015). This raises the possibility that urban environments function as modern analogues of previously exploited marginal or heterogeneous habitats (Russo and Ancillotto 2015; Vlaschenko et al. 2023). Species‐level traits have undoubtedly facilitated the successful colonisation of cities, as reflected by the high current abundance of *C. serotinus* among urban bat aggregations (Prylutska et al. 2023). However, our results suggest that sexual dimorphism may play an additional role, enabling the species to exploit contemporary heterogeneous landscapes through sex‐specific spatial niche partitioning (Ruckstuhl and Neuhaus 2005).

The sensitivity analysis confirmed that the documented sexual dimorphism is not an artefact of including seasonally captured immature individuals, with the direction and magnitude of dimorphism remaining consistent across all three sampling subsets (Table S5). This robustness reflects the early postnatal completion of the skeletal dimensions driving the observed dimorphism.

## 5 Conclusions

In conclusion, our morphometric analysis reveals pronounced female‐biased sexual dimorphism in *C. serotinus* , with wing surface areas showing the greatest differentiation (14%–15%), followed by mandibular dimensions (2%–3%) and forearm length (3.2%). The differential effect sizes across morphological systems—largest for wings, moderate for mandibles, smaller for other cranial structures—suggest that multiple selective pressures have shaped dimorphism, with flight performance during pregnancy likely representing the strongest driver. The pattern of larger females with enhanced wing areas but conserved wing shape is consistent with the Big Mother and flight load hypotheses, supporting the interpretation that dimorphism primarily compensates for pregnancy‐related mass increases. The pronounced dimorphism in mandibular structures related to bite force generation, combined with the documented spatial segregation in this population, provides morphological evidence supporting the hypothesis that sexual dimorphism enables intraspecific resource partitioning in this widespread synanthropic species. These findings underscore the importance of considering sex‐specific morphology and ecology in studies of bat populations, particularly in urban environments where males and females may experience different selective pressures.

## Author Contributions

**Anton Vlaschenko:** conceptualization (lead), data curation (supporting), funding acquisition (lead), investigation (supporting), methodology (supporting), project administration (lead), resources (lead), supervision (lead), validation (equal), visualization (supporting), writing – original draft (lead), writing – review and editing (equal). **Maryna Yerofieieva:** conceptualization (equal), data curation (lead), investigation (lead), project administration (equal), software (lead), writing – original draft (equal), writing – review and editing (equal). **Olena Holovchenko:** methodology (equal), resources (equal), writing – review and editing (equal). **Dina K. N. Dechmann:** conceptualization (equal), funding acquisition (lead), project administration (equal), writing – original draft (equal), writing – review and editing (equal).

## Funding

This research was conducted as part of the research activity of the UBRC, funded by the International Charitable Foundation ‘Oleksandr Feldman Foundation’ until 2024, with additional support from DierenPark Amersfoort Wildlife Fund (2022–2025), Future for Nature (2022) and International Foundation of Animal Welfare (2022–2024). The manuscript was finalised during the Philipp Schwartz Initiative Fellowship of A.V. at the Leibniz Institute for Zoo and Wildlife Research (Germany). Earlier stages of this work were realised by M.Y. with support of (i) the ‘Ukrainian Scientists in Danger’ programme (2022–2023) at the Leibniz Institute for Zoo and Wildlife Research (2022–2023), (ii) DAAD Eastern Partnerships Program short‐term scholarship for Ukrainian students (2022–2023) and (iii) ‘Ukrainian Researchers at Risk’ special scholarship from the Max Planck Institute of Animal Behaviour (April–October 2022).

## Ethics Statement

Craniometric and wing morphometric measurements were obtained from specimens from the Bat Carcasses Storing Collection of the Ukrainian Bat Rehabilitation Center (UBRC). Forearm length data were collected during routine intake procedures at the UBRC as part of the centre's long‐term monitoring programme. All procedures were conducted at the Ukrainian Bat Rehabilitation Center under the general permission of the Kharkiv Oblast Authority of Ecology and Natural Resources. No animals were captured or handled specifically for this study.

## Conflicts of Interest

The authors declare no conflicts of interest.

## Supporting information


**Figure S1:** Wing morphometric measurements of *C. serotinus* , illustrated on a postmortem specimen photographed in standardised ventral position with the left wing spread. (a) Linear wing dimensions: red lines indicate wing length (WL), arm wing length (AL), hand wing length (HL) and forearm length (Fa); green lines indicate metacarpal (MC3, MC4, MC5) and digit lengths (DG3, DG4, DG5). (b) Wing surface areas: total wing area (WA, outer polygon), subdivided into hand wing area (HA) and arm wing area (AA). (c) Skeletal elements and landmarks (numbered 1–13) used for digitisation in ImageJ: individual phalanges of digits III–V (PP—proximal phalanx, MP—medial phalanx, DP—distal phalanx), metacarpals (MC) and forearm (Fa). All measurements were taken from digitised photographs using ImageJ software only the left wing was used for analysis.
**Table S1:** Raw craniometric measurements of *C. serotinus* skulls (*n* = 137). All cranial measurements in mm; Ra (forearm length) in mm. Date—day of the month of admission to the UBRC; Month—month of admission. Ra—forearm length; Brain—braincase breadth; Z‐Z—zygomatic width; B‐B—interorbital width; Mg‐Mg—mastoid width; Cs‐Cs—canine width; Cs‐Mg—upper toothrow length; Pr‐La—greatest skull length; A‐Sq—zygomatic arch length; Shpb—skull height; In‐Cn—total mandible length; Ci‐Mi—lower toothrow length; Ci‐Co—canine‐coronoid distance; Co‐Cn—condyle‐coronoid distance; Co‐R—coronoid height; Pr‐Fm—condylobasal length. Sex: F—female, M—male. Age: Ad—adult, Sad—subadult. Missing values: ‘—’.
**Table S2:** Wing morphometric measurements of *C. serotinus* (*n* = 22). Linear measurements in cm, areas in cm^2^. Fa—forearm length; MC2–MC5—metacarpal lengths; PP3–PP5—proximal phalanx lengths; MP3—medial phalanx of digit 3; DP3–DP5—distal phalanx lengths; DG3–DG5—total digit lengths; WL—wing length; HL—hand‐wing length; AL—arm‐wing length; WA—total wing area; HA—hand‐wing area; AA—arm‐wing area. Sex: F—female, M—male. Age: AD—adult, SD—subadult.
**Table S3:** Results of two‐way ANOVA (Sex × Age) for craniometric parameters of *C. serotinus* . Only specimens with known age were included (*n* = 97). *F*—*F*‐statistic; *p*—*p*‐value; *η* ^2^—partial eta‐squared (effect size); Int—Sex × Age interaction. Significance: *p < 0.05, **p < 0.01, ***p < 0.001.
**Table S4:** Results of two‐way ANOVA (Sex × Age) for wing morphometric parameters of *C. serotinus* (*n* = 22). *F*—*F*‐statistic; *p*—*p*‐value; *η* ^2^—partial eta‐squared (effect size); Int—Sex × Age interaction. Significance: *p < 0.05, **p < 0.01, ***p < 0.001.
**Table S5:** Sensitivity analysis of sexual dimorphism in cranial and forearm dimensions of *Cnephaeus serotinus* under three sampling scenarios. Comparison of sexual dimorphism estimates between (1) the full sample, (2) the sample excluding August subadults and August individuals of unknown age (i.e., specimens most likely to be this‐year‐born with incomplete cranial ossification) and (3) confirmed adults only. Welch's *t*‐test was used for between‐sex comparisons; *p*‐values were adjusted for multiple testing using the Benjamini–Hochberg FDR procedure. Cohen's *d* > 0 indicates female‐biased dimorphism. Significant comparisons after FDR correction (*p* < 0.05) are marked with an asterisk and shown in bold.
**Table S6:** Comparison of craniometric and forearm dimensions between August‐collected immature individuals of *C. serotinus* and conspecifics of comparable age collected in other months. Three independent comparisons were performed: (1) August subadults vs. subadults from other months; (2) August individuals of unknown age vs. unknown‐age individuals from other months; (3) August immature + unknown‐age individuals (pooled) vs. corresponding pooled subset from other months. Between‐group comparisons used Welch's *t*‐test (parametric) and Mann–Whitney *U* test (non‐parametric); effect sizes are reported as Cohen's *d*, where *d* < 0 indicates that August individuals are smaller. Significant differences (*p* < 0.05) are marked with an asterisk and shown in bold.

## Data Availability

All data supporting the findings of this study are provided as Supporting Information accompanying this article (Tables S1–S6).
