# Supplementary material for: Sexual Dimorphism as a Potential Driver of Trophic Specialisation in a Sedentary Synanthropic Bat Species
Source: Ecol Evol. 2026 Aug 16;16(8):e74202. doi: 10.1002/ece3.74202 (PMC13477827; doi:10.1002/ece3.74202)
Supplement: Supplementary file 1 [file ECE3-16-e74202-s001.docx]

**Supplementary Materials**

Yerofieieva M., Holovchenko O., Dechmann D.K. N., Vlaschenko A.

**Sexual dimorphism as a potential driver of trophic specialisation in a sedentary synanthropic bat species**

| a   | b | c |
| --- | --- | --- |
| **Fig. S1** Wing morphometric measurements of *C. serotinus*, illustrated on a postmortem specimen photographed in standardized ventral position with the left wing spread. **(a)** Linear wing dimensions: red lines indicate wing length (WL), arm wing length (AL), hand wing length (HL), and forearm length (Fa); green lines indicate metacarpal (MC3, MC4, MC5) and digit lengths (DG3, DG4, DG5). **(b)** Wing surface areas: total wing area (WA, outer polygon), subdivided into hand wing area (HA) and arm wing area (AA). **(c)** Skeletal elements and landmarks (numbered 1–13) used for digitization in ImageJ: individual phalanges of digits III–V (PP — proximal phalanx, MP — medial phalanx, DP — distal phalanx), metacarpals (MC), and forearm (Fa). All measurements were taken from digitized photographs using ImageJ software only the left wing was used for analysis. | | |

**Table S1.** Raw craniometric measurements of *C. serotinus* skulls (n = 137). All cranial measurements in mm; Ra (forearm length) in mm. Date — day of the month of admission to the UBRC; Month — month of admission. Abbreviations: Ra — forearm length; Brain — braincase breadth; Z-Z — zygomatic width; B-B — interorbital width; Mg-Mg — mastoid width; Cs-Cs — canine width; Cs-Mg — upper toothrow length; Pr-La — greatest skull length; A-Sq — zygomatic arch length; Shpb — skull height; In-Cn — total mandible length; Ci-Mi — lower toothrow length; Ci-Co — canine-coronoid distance; Co-Cn — condyle-coronoid distance; Co-R — coronoid height; Pr-Fm — condylobasal length. Sex: F — female, M — male. Age: Ad — adult, Sad — subadult. Missing values: "—".

| **№** | **Sex** | **Age** | **Date** | **Month** | **Year** | **Ra** | **Brain** | **Z-Z** | **B-B** | **Mg-Mg** | **Cs-Cs** | **Cs-Mg** | **Pr-La** | **A-Sq** | **Shpb** | **In-Cn** | **Ci-Mi** | **Ci-Co** | **Co-Cn** | **Co-R** | **Pr-Fm** |
| --- | --- | --- | --- | --- | --- | --- | --- | --- | --- | --- | --- | --- | --- | --- | --- | --- | --- | --- | --- | --- | --- |
| ES-95 | F | — | 14 | August | 2006 | 53.00 | — | 14.06 | 4.23 | 8.38 | — | — | — | — | — | 15.88 | — | — | 5.33 | 6.62 | — |
| ES-251 | M | Sad | 19 | November | 2016 | 52.20 | 9.60 | 13.87 | 4.23 | 8.49 | 6.43 | 7.40 | 20.21 | 10.05 | 6.60 | 15.21 | 8.29 | 10.70 | 5.16 | 5.90 | 19.71 |
| ES-249 | M | Sad | 3 | April | 2018 | 51.60 | 9.53 | 13.74 | 4.56 | 8.45 | 6.23 | 7.31 | 20.57 | 9.93 | 6.83 | 15.20 | 8.29 | 11.36 | 4.74 | 5.44 | 19.90 |
| ES-248 | M | Sad | 16 | January | 2018 | 51.90 | 9.45 | 13.86 | 4.45 | 8.24 | 6.43 | 7.49 | 20.68 | 10.70 | 6.30 | 15.25 | 8.67 | 11.23 | 5.00 | 6.04 | 19.94 |
| ES-247 | M | Sad | 15 | December | 2016 | 52.40 | 9.60 | — | 4.80 | 8.91 | 6.43 | 7.71 | 20.37 | 10.76 | 6.77 | 15.61 | 8.45 | 11.84 | 5.01 | 5.96 | 20.10 |
| ES-246 | F | Sad | 3 | August | 2017 | 52.60 | 9.28 | 13.76 | 4.18 | 8.83 | 6.21 | 7.40 | 20.25 | 10.19 | 6.41 | 15.24 | 8.46 | 11.41 | 5.10 | 6.23 | 19.62 |
| ES-245 | F | Ad | 12 | July | 2018 | 54.10 | 9.17 | — | 4.39 | 8.62 | 6.52 | 7.65 | 20.80 | 10.21 | 6.62 | 15.56 | 8.57 | 11.14 | 4.95 | 5.48 | 19.84 |
| ES-244 | M | Sad | 25 | February | 2018 | 53.50 | 9.80 | 13.87 | 4.51 | — | — | 7.76 | 21.70 | 10.79 | 6.21 | 15.95 | 8.57 | — | — | — | 20.40 |
| ES-243 | M | Sad | 6 | March | 2018 | 52.40 | 9.34 | — | 4.36 | 8.60 | 6.42 | 7.27 | 20.03 | 9.86 | 6.30 | 14.89 | 8.34 | 10.94 | 4.87 | 5.73 | 19.21 |
| ES-242 | M | Sad | 21 | January | 2013 | 47.40 | 9.13 | 13.46 | 4.11 | 8.29 | 6.05 | 7.34 | 19.89 | 10.02 | 6.23 | 15.03 | 8.22 | 10.91 | 5.07 | — | 19.44 |
| ES-241 | F | — | 19 | April | 2011 | — | 9.48 | 14.36 | 4.47 | — | 6.71 | 7.92 | 21.44 | 10.58 | 6.35 | 16.23 | — | 12.25 | 5.29 | 6.37 | 21.01 |
| ES-240 | F | Sad | 7 | April | 2016 | 53.90 | 9.36 | 14.14 | 4.36 | 8.62 | 6.30 | 7.65 | 21.34 | 9.96 | 6.60 | 15.64 | 8.47 | 11.78 | 5.06 | 6.06 | 20.32 |
| ES-239 | F | Sad | 16 | January | 2011 | 51.50 | 9.59 | 14.11 | 4.31 | 8.43 | 6.45 | 7.74 | 21.26 | 10.25 | 6.61 | 15.72 | 8.62 | 11.39 | 5.24 | 5.97 | 20.40 |
| ES-237 | M | Sad | 18 | January | 2017 | 51.90 | 9.50 | 13.67 | 4.58 | 8.46 | 6.62 | 7.71 | 20.78 | 10.13 | 6.75 | 15.53 | 8.96 | 11.43 | 4.79 | 5.93 | 20.02 |
| ES-235 | M | Sad | 18 | February | 2018 | 52.40 | 9.42 | 13.72 | 4.25 | 8.33 | 6.13 | 7.39 | 20.86 | 10.14 | 6.69 | — | 8.37 | 11.58 | 5.02 | 5.75 | 20.08 |
| ES-234 | M | Ad | 31 | August | 2008 | 53.60 | 9.52 | 14.41 | 4.49 | 8.69 | 6.67 | 7.59 | 20.79 | 10.21 | 6.67 | 15.76 | 8.64 | 11.64 | 5.02 | 5.70 | 19.78 |
| ES-233 | F | Sad | 29 | August | 2005 | 53.50 | 9.62 | 14.36 | 4.42 | 8.70 | 6.67 | 7.55 | 20.89 | 10.49 | 6.45 | 15.46 | 8.62 | 12.40 | 5.14 | 6.43 | 20.16 |
| ES-232 | F | — | 14 | March | 2011 | 53.00 | — | — | 4.78 | 8.97 | — | 7.61 | — | 10.65 | — | 15.83 | 8.50 | 11.81 | 5.18 | 6.06 | — |
| ES-231 | M | — | 15 | January | 2005 | 52.70 | 10.07 | 14.38 | 4.65 | 8.65 | 6.38 | 7.36 | 20.40 | 10.16 | 6.69 | 14.96 | 8.38 | — | — | — | 19.64 |
| ES-230 | M | Ad | 19 | December | 2012 | 53.00 | 9.27 | 13.87 | 4.46 | 8.27 | 6.33 | 7.46 | 20.87 | 10.06 | 7.09 | 15.46 | 8.66 | 10.85 | 5.33 | 5.84 | 19.98 |
| ES-228 | F | Sad | 3 | August | 2017 | 53.30 | 9.55 | 13.07 | 4.38 | 8.27 | 6.00 | 7.27 | — | 10.06 | — | 14.72 | 8.15 | 10.66 | 4.82 | 5.47 | — |
| ES-227 | F | Sad | 29 | November | 2017 | 53.10 | 9.86 | 14.15 | 4.54 | 8.35 | 6.69 | 7.53 | 20.36 | 9.89 | 6.52 | 15.39 | 8.50 | 11.31 | 5.03 | 5.65 | 19.20 |
| ES-226 | F | Sad | 16 | November | 2018 | 53.80 | 9.33 | 14.06 | 4.49 | 8.86 | 7.00 | 7.86 | — | 10.64 | 7.06 | 16.06 | 8.69 | 11.97 | 5.49 | 6.51 | 20.22 |
| ES-225 | F | — | 15 | August | 2017 | — | 9.99 | — | 4.21 | 8.05 | 6.37 | 7.19 | — | 10.50 | — | 15.62 | 8.64 | — | — | — | — |
| ES-224 | F | Sad | 12 | January | 2017 | 50.90 | 9.80 | 14.09 | 4.31 | 8.36 | 6.48 | 7.60 | 20.45 | 10.42 | 6.81 | 15.46 | 8.54 | — | — | — | 19.70 |
| ES-223 | M | Sad | 7 | February | 2018 | 52.70 | 9.57 | 13.86 | 4.34 | 8.61 | 6.05 | 7.28 | 20.07 | 9.97 | 6.51 | 14.76 | 8.27 | 11.23 | 4.81 | 5.87 | 19.21 |
| ES-222 | M | Sad | 4 | September | 2016 | 49.30 | 9.52 | 14.29 | 4.66 | 8.40 | 6.34 | 7.17 | 20.51 | 9.97 | 6.76 | 15.17 | 8.13 | 11.37 | 4.98 | 5.82 | 19.60 |
| ES-221 | F | Sad | 5 | January | 2016 | 52.90 | 9.42 | 13.86 | 4.45 | 8.91 | 6.56 | 7.57 | — | 10.04 | 6.88 | 15.65 | 8.74 | 11.45 | 5.25 | 6.16 | 19.85 |
| ES-220 | M | Sad | 15 | December | 2017 | 52.70 | 9.67 | 14.55 | 4.40 | 8.53 | 6.39 | 7.50 | 20.95 | 10.37 | 7.09 | 15.55 | 8.45 | 11.61 | 5.08 | 6.03 | 19.98 |
| ES-219 | M | Sad | 4 | August | 2017 | 53.30 | 9.62 | 13.87 | 4.39 | 8.32 | 6.22 | 7.50 | 20.77 | 10.30 | 6.70 | 15.41 | 8.44 | 11.49 | 5.02 | 6.14 | 20.00 |
| ES-218 | F | Sad | 30 | September | 2010 | 51.70 | 9.59 | 13.75 | 4.53 | 8.30 | 6.37 | 7.62 | 20.39 | 9.91 | 6.41 | 14.79 | 8.69 | 11.50 | 4.52 | 5.77 | 19.65 |
| ES-217 | F | Sad | 15 | December | 2018 | — | 9.48 | 14.66 | 4.56 | 8.86 | 6.65 | 7.54 | — | 10.62 | — | 15.58 | 8.65 | — | — | — | 20.14 |
| ES-216 | F | Sad | 20 | November | 2018 | 52.80 | 9.77 | 14.11 | 4.30 | 8.62 | 6.79 | 7.54 | 21.08 | 10.37 | 6.63 | 15.83 | 8.54 | 11.53 | 5.46 | 6.30 | 19.88 |
| ES-215 | M | Sad | 27 | November | 2009 | 55.00 | 9.58 | 13.83 | 4.29 | 8.25 | 6.37 | 7.55 | 21.09 | 10.64 | 6.56 | 15.93 | 8.69 | — | — | — | 20.50 |
| ES-214 | M | Sad | 25 | June | 2011 | — | 9.34 | 13.87 | 4.74 | 8.29 | 6.65 | 7.49 | 20.89 | 10.09 | 6.85 | — | 8.50 | 11.07 | 5.04 | 5.70 | 20.03 |
| ES-213 | M | Sad | 12 | January | 2017 | 51.90 | 9.83 | 13.88 | 4.38 | 8.39 | 6.53 | 7.65 | 21.14 | 10.12 | — | 15.45 | 8.65 | 11.39 | 4.94 | 5.92 | — |
| ES-212 | F | Sad | 5 | March | 2017 | 53.60 | 9.74 | 14.27 | 4.58 | 8.74 | 6.41 | 7.57 | 21.13 | 10.68 | 6.69 | 15.84 | 8.48 | — | — | — | 20.19 |
| ES-211 | M | Ad | 16 | January | 2017 | 51.20 | — | 13.83 | 4.58 | 8.61 | 6.29 | 7.47 | — | 10.28 | 6.86 | 15.41 | 8.30 | — | — | — | 20.01 |
| ES-209 | F | Sad | 4 | January | 2018 | 53.50 | 9.98 | 14.73 | 4.53 | 8.92 | 6.67 | 7.66 | 20.95 | 10.15 | 6.83 | 15.50 | 8.53 | 11.42 | 5.21 | 6.40 | 19.76 |
| ES-208 | M | Sad | un | December | 2009 | 53.70 | 9.37 | 15.23 | 4.31 | 8.32 | 6.41 | 7.28 | — | 10.07 | — | — | — | — | — | — | 19.51 |
| ES-207 | M | Sad | 24 | November | 2017 | 50.00 | 9.19 | 13.76 | 4.26 | 8.35 | 6.17 | 7.23 | 20.09 | 10.16 | 6.53 | 15.18 | 8.19 | 11.16 | 5.12 | 5.99 | 19.65 |
| ES-206 | F | Sad | 18 | January | 2008 | 52.20 | 9.36 | 14.67 | 4.20 | 8.66 | 6.51 | 7.39 | 19.95 | 9.87 | 6.67 | 15.18 | 8.36 | 11.23 | 5.24 | 6.19 | 19.27 |
| ES-204 | M | Ad | 12 | June | 2009 | 52.00 | 9.52 | 13.89 | 5.12 | 8.12 | 6.25 | 7.41 | 20.40 | 10.01 | 6.74 | 15.17 | 8.33 | 11.30 | 4.93 | 5.79 | 19.52 |
| ES-203 | M | Ad | 24 | February | 2011 | 53.50 | 9.46 | 14.72 | 4.55 | 9.07 | 6.81 | 7.95 | 20.68 | 10.22 | 6.69 | 15.78 | 8.93 | 12.13 | 4.99 | 6.23 | 20.20 |
| ES-202 | M | — | un | December | 2010 | — | 9.55 | 14.31 | 4.48 | 8.31 | 6.63 | 7.65 | 21.46 | 10.62 | 7.05 | 16.01 | 8.55 | 11.97 | 5.13 | 6.16 | 20.81 |
| ES-201 | F | — | un | April | 2006 | — | 9.52 | 14.25 | 4.59 | 8.76 | 6.60 | 7.64 | — | 10.50 | 7.06 | 15.65 | 8.53 | 11.62 | 5.16 | 6.09 | — |
| ES-200 | M | Sad | 21 | February | 2011 | 52.90 | 9.62 | 14.13 | 4.36 | 8.48 | 6.69 | 7.58 | 20.70 | 10.24 | 6.57 | 15.53 | 8.47 | 11.47 | 5.09 | 6.00 | 20.15 |
| ES-199 | M | Ad | 3 | May | 2012 | 50.80 | 9.68 | 14.14 | 4.60 | 8.50 | 6.82 | 7.54 | 20.92 | 10.23 | 6.87 | 15.34 | 8.66 | 11.47 | 4.96 | 5.96 | 19.67 |
| ES-198 | M | Sad | 4 | September | 2006 | 49.20 | 9.87 | 14.54 | 4.65 | 8.51 | 6.47 | 7.47 | — | 10.36 | — | 15.47 | 8.38 | 11.52 | 5.09 | 5.97 | 20.32 |
| ES-195 | F | Sad | 22 | January | 2009 | 52.40 | 9.76 | 14.07 | 4.55 | 8.63 | 6.70 | 7.44 | — | 10.18 | 7.08 | 15.54 | 8.46 | 11.48 | 4.97 | 5.99 | 19.29 |
| ES-194 | M | — | 14 | January | 2009 | 51.50 | 9.49 | 14.13 | 4.33 | 8.80 | 6.62 | 7.59 | 20.58 | 10.25 | 6.66 | 15.60 | 8.43 | 11.22 | 5.24 | 5.89 | 19.89 |
| ES-193 | M | — | 10 | February | 2009 | 51.20 | 9.61 | 14.26 | 4.28 | 8.94 | 6.70 | 7.52 | 20.75 | 10.32 | 6.62 | — | 8.62 | 11.32 | 5.08 | 5.89 | 19.99 |
| ES-192 | M | — | 10 | August | 2006 | 51.00 | 9.40 | 13.52 | 4.36 | 8.49 | 6.37 | 7.43 | 20.57 | 10.10 | 6.24 | 15.23 | 8.35 | 11.28 | 4.74 | 5.63 | 19.77 |
| ES-191 | M | Ad | 13 | January | 2014 | 53.90 | 9.66 | 14.51 | 4.65 | 8.77 | 6.61 | 7.66 | 21.08 | 10.01 | 6.90 | 15.73 | 8.82 | 12.04 | 4.99 | 5.94 | 20.18 |
| ES-190 | M | Ad | un | December | 2013-14 | — | 9.89 | 14.05 | 4.44 | 8.49 | 6.42 | 7.40 | 20.99 | 10.37 | 6.75 | 15.53 | 8.43 | 11.22 | 5.20 | 5.77 | 20.31 |
| ES-189 | F | — | 12 | December | 2013 | 56.20 | 9.52 | 13.76 | 4.46 | 8.53 | 6.45 | 7.54 | — | 10.09 | 7.04 | 15.37 | 8.57 | 11.07 | 5.09 | 6.10 | 20.00 |
| ES-188 | F | — | 27 | October | 2006 | 52.30 | 9.40 | 13.56 | 4.45 | 8.40 | 6.59 | 7.56 | — | 10.11 | — | 15.76 | 8.62 | 11.42 | 5.33 | 6.01 | 19.88 |
| ES-187 | M | — | 17 | October | 2018 | — | 9.63 | 13.77 | 4.47 | 8.67 | — | — | — | 10.06 | 6.40 | 15.28 | 8.41 | 11.24 | 5.00 | 5.86 | — |
| ES-184 | F | — | un | December | 2016 | — | 9.61 | 14.42 | 4.14 | 8.63 | 6.50 | 7.72 | 21.06 | 10.66 | 6.93 | 16.03 | 8.56 | — | — | — | 20.48 |
| ES-183 | F | Ad | 8 | December | 2016 | 54.70 | 9.38 | 14.50 | 4.55 | 8.76 | 6.82 | 7.86 | 21.38 | 10.33 | 6.63 | 15.96 | 8.97 | 11.74 | 5.52 | 6.46 | 20.37 |
| ES-182 | F | — | 28 | January | 2017 | — | — | 14.02 | 4.34 | 8.42 | 6.18 | 7.64 | — | 10.52 | — | 16.00 | 8.51 | 11.71 | 5.36 | 6.13 | — |
| ES-181 | M | Ad | 7 | December | 2016 | 50.30 | 9.26 | 13.50 | 4.45 | 8.34 | 6.32 | 7.53 | 20.58 | 10.08 | 6.48 | 15.44 | 8.57 | 11.73 | 4.91 | 5.70 | 19.61 |
| ES-180 | F | Sad | 20 | July | 2016 | 47.40 | — | — | 4.30 | — | — | — | — | 9.63 | — | — | 8.64 | — | — | — | — |
| ES-179 | F | Sad | 30 | December | 2014 | 52.70 | 9.44 | 13.81 | 4.22 | 8.33 | 6.21 | 7.40 | 20.73 | 10.15 | 6.46 | 15.28 | 8.27 | 11.33 | 4.82 | 5.90 | 19.92 |
| ES-178 | M | — | 26 | January | 2015 | — | 9.62 | 14.08 | 4.56 | 8.72 | 5.98 | 7.37 | 20.39 | 10.16 | 6.66 | 15.28 | 8.35 | 11.36 | 4.99 | 5.88 | 19.81 |
| ES-177 | M | — | 26 | August | 2008 | — | — | 13.80 | 4.64 | 8.40 | 6.37 | 7.37 | — | 9.97 | — | 15.23 | 8.33 | 11.07 | 4.91 | 5.84 | — |
| ES-176 | F | Sad | 6 | February | 2015 | 52.20 | 9.10 | 13.90 | 4.35 | 8.74 | 6.61 | 7.73 | 20.07 | 10.44 | 6.45 | 15.70 | 8.61 | 11.36 | 5.08 | 5.99 | 19.90 |
| ES-175 | F | Sad | 24 | January | 2015 | 55.10 | 9.83 | 14.96 | 4.31 | 8.76 | 6.81 | 7.77 | 21.46 | 10.68 | 6.76 | 16.09 | 8.78 | 12.04 | 5.36 | 6.39 | 20.86 |
| ES-174 | F | Ad | 6 | February | 2015 | 52.40 | 9.86 | 14.59 | 4.36 | 8.87 | 6.46 | 7.71 | 21.39 | 10.44 | 6.71 | 15.86 | 8.76 | 12.34 | 5.15 | 6.26 | 20.55 |
| ES-173 | M | — | 10 | August | 2006 | 51.00 | 9.19 | — | 4.22 | 8.29 | 6.14 | 7.20 | — | 10.07 | 6.31 | 15.04 | 8.13 | 11.13 | 4.71 | 5.60 | 19.26 |
| ES-172 | M | — | 10 | August | 2006 | 51.80 | — | 13.49 | 4.47 | 8.48 | 6.50 | 7.40 | — | 9.85 | — | 14.04 | 8.49 | 11.08 | 4.93 | 5.86 | — |
| ES-171 | F | Ad | 6 | May | 2004 | 53.50 | 9.33 | 14.27 | 4.40 | 8.87 | 6.62 | 7.69 | 21.29 | 10.62 | 6.62 | 15.78 | 8.75 | 11.53 | 5.37 | 6.30 | 20.29 |
| ES-170 | F | Ad | 26 | August | 2008 | — | 9.61 | — | 4.22 | 8.37 | 6.43 | 7.54 | 21.00 | 10.74 | — | 16.05 | 8.68 | 11.72 | 5.38 | 6.00 | 20.06 |
| ES-169 | M | — | 26 | August | 2008 | — | 9.26 | 13.41 | 4.13 | 8.30 | 6.55 | 7.47 | 20.23 | 9.98 | 6.85 | 15.16 | 8.32 | 11.03 | 5.20 | 6.04 | 19.67 |
| ES-168 | M | — | 26 | August | 2008 | — | 9.35 | 13.96 | 4.28 | 8.77 | 6.41 | 7.50 | 20.27 | 9.80 | — | 15.15 | 8.51 | 11.41 | 4.77 | 5.88 | — |
| ES-167 | M | — | 26 | August | 2008 | — | 9.40 | — | 4.58 | 8.30 | 5.96 | 7.45 | 20.17 | — | 6.70 | 15.13 | 8.32 | 10.85 | 4.71 | 5.30 | 19.55 |
| ES-166 | M | Ad | 31 | May | 2005 | 52.00 | 9.28 | — | 4.21 | 8.69 | 6.45 | 7.64 | — | 10.32 | — | 15.38 | 8.45 | — | — | — | 20.17 |
| ES-164 | F | Ad | 18 | December | 2015 | 53.80 | 9.81 | 14.60 | 4.33 | 8.66 | 6.45 | 7.81 | 20.19 | 10.60 | 6.87 | 16.37 | 8.84 | 12.29 | 5.67 | 6.50 | 20.98 |
| ES-163 | F | Ad | 18 | January | 2016 | 56.90 | 9.89 | 14.73 | 4.51 | 8.75 | 6.52 | 7.58 | 21.72 | 10.66 | 7.02 | 16.18 | 8.66 | 12.09 | 5.41 | 6.15 | 20.90 |
| ES-162 | F | Ad | 4 | January | 2016 | 53.00 | 9.74 | 14.81 | 4.19 | 8.90 | 6.63 | 7.60 | 20.90 | 10.75 | 7.03 | 15.74 | 8.47 | 11.70 | 5.28 | 6.32 | 20.38 |
| ES-161 | M | — | 31 | December | 2015 | 53.60 | 8.87 | 14.34 | 4.53 | 8.84 | 6.58 | 7.90 | 21.36 | 10.23 | 6.59 | 15.54 | 8.80 | 11.53 | 5.01 | 5.88 | 20.07 |
| ES-160 | M | Sad | 25 | January | 2016 | 54.50 | 9.53 | 14.31 | 4.53 | 8.69 | 6.53 | 7.52 | 21.13 | 10.61 | 7.20 | 15.65 | 8.58 | 11.70 | 5.04 | 5.91 | 20.31 |
| ES-159 | F | Ad | 7 | September | 2015 | 52.90 | 9.31 | 13.43 | 4.23 | — | 6.39 | — | — | 10.41 | 6.68 | 15.71 | 8.45 | 11.64 | 5.06 | 5.91 | 20.40 |
| ES-158 | F | Sad | 19 | January | 2016 | 53.90 | 9.65 | 14.85 | 4.23 | 8.77 | 6.50 | 7.57 | 20.69 | 10.18 | 6.85 | 15.78 | 8.61 | 11.11 | 5.15 | 5.69 | 20.25 |
| ES-157 | M | Ad | 17 | December | 2015 | 51.90 | 9.77 | — | 4.51 | 8.36 | 6.61 | 7.61 | 21.18 | 10.26 | 7.02 | — | — | — | — | — | 20.17 |
| ES-156 | F | Ad | 10 | February | 2016 | 53.20 | 9.97 | 14.41 | 4.36 | 8.73 | 6.70 | 7.74 | 21.51 | 10.48 | 6.98 | 15.83 | 8.76 | 11.89 | 5.02 | 5.97 | 20.59 |
| ES-155 | M | — | 28 | January | 2016 | 49.30 | 9.03 | 13.47 | 4.22 | 8.65 | 6.25 | 7.23 | 19.64 | 9.76 | 6.25 | 14.80 | 8.08 | 10.92 | 4.80 | 5.69 | 18.79 |
| ES-153 | F | — | 10 | August | 2005 | 54.40 | 9.73 | 14.48 | 4.67 | 8.70 | 6.64 | 7.64 | — | 10.60 | — | 15.81 | 8.50 | — | — | — | 20.21 |
| ES-152 | F | Ad | 22 | March | 2004 | 53.20 | 9.58 | 13.82 | 4.63 | 8.30 | 6.37 | 7.51 | 20.67 | 10.29 | 6.92 | 15.64 | 8.36 | 11.26 | 5.21 | 5.99 | 20.20 |
| ES-151 | F | Ad | 6 | March | 2008 | 53.80 | 9.72 | 14.53 | 4.44 | 8.86 | 6.65 | 7.61 | — | 10.37 | 6.87 | — | 8.64 | 11.64 | 5.15 | 6.22 | — |
| ES-150 | M | Sad | 23 | February | 2005 | 53.00 | 9.55 | 14.15 | 4.25 | 8.52 | 6.59 | 7.46 | — | 9.76 | — | 15.23 | 8.58 | 11.61 | 4.66 | 5.75 | — |
| ES-149 | F | — | 26 | August | 2008 | — | 9.54 | 14.43 | 4.57 | 8.54 | 6.79 | 7.87 | 21.66 | 10.36 | 7.24 | 15.99 | 8.96 | 12.39 | 5.23 | 6.23 | 20.54 |
| ES-148 | M | Ad | 31 | May | 2008 | 51.70 | 9.78 | 14.23 | 4.37 | 8.64 | 6.41 | 7.31 | 20.67 | 10.18 | 7.27 | 15.35 | 8.19 | 11.56 | 4.96 | 5.88 | 19.90 |
| ES-147 | M | — | 26 | August | 2008 | — | 9.43 | 13.81 | 4.15 | 8.59 | 6.34 | 7.50 | 20.97 | 10.22 | 6.70 | — | 8.48 | 11.49 | 4.77 | 5.83 | 20.19 |
| ES-146 | F | — | 26 | August | 2008 | — | 9.50 | — | 4.46 | 8.48 | 6.53 | 7.32 | — | 10.15 | — | 15.11 | 8.54 | 10.84 | 5.24 | 6.03 | — |
| ES-145 | F | Sad | 27 | December | 2006 | 52.90 | 9.12 | 14.03 | 4.32 | 8.31 | 6.34 | 7.20 | 20.10 | 10.12 | 6.82 | 15.13 | 8.22 | 10.87 | 4.92 | 5.75 | 19.41 |
| ES-143 | F | — | 10 | August | 2008 | 50.60 | 9.32 | 13.99 | 4.45 | 8.63 | 6.49 | 7.41 | 20.31 | 9.83 | 6.65 | 14.93 | 8.13 | 11.21 | 4.81 | 5.92 | 19.28 |
| ES-142 | M | — | 26 | August | 2016 | — | — | — | 4.09 | 8.35 | 6.22 | 7.55 | — | 10.22 | — | 15.24 | 8.39 | 11.54 | 4.82 | 5.82 | — |
| ES-141 | F | Ad | 12 | January | 2013 | 52.40 | 9.31 | 14.31 | 4.40 | 8.91 | 6.52 | 7.66 | 21.14 | 10.56 | 6.68 | 15.83 | 8.56 | — | — | — | 20.22 |
| ES-140 | M | Ad | 19 | January | 2008 | 52.40 | 9.69 | 14.36 | 4.34 | 8.54 | 6.73 | 7.64 | 20.85 | 9.96 | 6.95 | 15.18 | 8.39 | 11.55 | 4.89 | 5.72 | 20.09 |
| ES-139 | M | — | 26 | August | 2008 | — | 9.36 | 13.84 | 4.19 | 8.57 | 6.28 | 7.47 | 20.69 | 10.26 | 6.82 | 15.26 | 8.59 | 11.54 | 4.74 | 5.90 | 19.81 |
| ES-138 | M | — | 7 | May | 2016 | 53.40 | 9.34 | 16.89 | 4.27 | 8.35 | 6.65 | 7.60 | 20.53 | 10.25 | 6.93 | 15.36 | 8.44 | 11.53 | 5.18 | 6.01 | 19.89 |
| ES-137 | F | Sad | 15 | February | 2013 | 50.40 | 9.01 | 13.15 | 4.35 | 8.23 | 6.25 | 7.04 | 19.38 | 9.47 | 6.63 | 14.69 | 8.00 | — | — | — | 18.81 |
| ES-136 | F | Sad | 24 | November | 2015 | — | 9.59 | 14.26 | 4.42 | 8.60 | 6.65 | 7.60 | 20.75 | 10.19 | 6.91 | 15.52 | 8.71 | — | — | — | 20.01 |
| ES-134 | M | Ad | 23 | December | 2015 | 51.00 | 9.49 | 14.11 | 4.51 | 8.81 | 6.17 | 7.37 | 20.86 | 10.10 | 6.68 | 15.23 | 8.45 | 11.65 | 5.12 | 6.11 | 19.92 |
| ES-133 | F | Ad | 23 | September | 2015 | 52.60 | 9.71 | 14.57 | 4.46 | 8.41 | 6.45 | 7.23 | 20.86 | 10.24 | 7.05 | 15.38 | 8.43 | 12.02 | 4.93 | — | 19.73 |
| ES-132 | F | Ad | 3 | February | 2016 | 53.20 | 9.72 | 14.47 | 4.53 | 8.93 | 6.40 | 7.49 | 20.82 | 10.46 | 7.11 | 15.63 | 8.46 | — | — | — | 20.07 |
| ES-131 | F | — | 30 | January | — | — | 9.35 | — | 4.44 | 8.74 | 6.54 | 8.00 | 21.34 | 10.70 | 7.07 | 16.03 | 9.00 | — | — | — | 20.53 |
| ES-130 | F | Sad | 6 | August | 2015 | 53.60 | 9.36 | — | 4.45 | 8.89 | 6.50 | 7.46 | — | 10.31 | — | 15.47 | 8.61 | 10.94 | 4.98 | 5.84 | 19.67 |
| ES-129 | F | Ad | 16 | March | 2016 | 55.40 | 9.49 | 14.32 | 4.32 | 9.00 | 6.84 | 7.95 | 21.38 | 10.71 | — | 15.97 | 8.83 | 12.42 | 5.00 | 5.83 | 20.61 |
| ES-128 | F | Sad | 3 | February | 2016 | 55.30 | 9.38 | 14.27 | 4.29 | 8.87 | 6.50 | 7.74 | 20.61 | 10.32 | 7.05 | 15.81 | 8.94 | 11.54 | 4.95 | 5.59 | 20.14 |
| ES-127 | M | — | 3 | October | 2015 | — | 9.51 | 14.22 | 4.47 | 8.54 | 6.72 | 7.63 | 20.82 | 10.15 | 6.62 | 15.40 | 8.58 | 11.42 | 5.21 | 5.94 | 19.97 |
| ES-126 | M | Ad | 24 | January | 2016 | 52.00 | 9.71 | 14.22 | 4.82 | 8.85 | 6.57 | 7.71 | 20.73 | 10.40 | 6.85 | 15.81 | 8.70 | — | — | — | 19.65 |
| ES-125 | F | Sad | 1 | January | 2016 | 53.50 | 9.67 | 14.35 | 4.34 | 8.54 | 6.74 | 7.80 | 21.21 | 10.47 | 6.80 | 15.80 | 8.69 | — | — | — | 20.36 |
| ES-124 | M | Sad | 2 | March | 2016 | 52.00 | 9.26 | 14.12 | 4.30 | 8.91 | — | 7.44 | — | 10.45 | — | 15.37 | 8.51 | — | — | — | 19.94 |
| ES-123 | M | Ad | 10 | January | 2016 | 52.20 | 9.72 | 14.31 | 4.60 | 8.81 | 6.74 | 7.70 | 20.96 | 10.38 | 6.67 | 15.64 | 8.64 | 11.75 | 5.24 | 6.09 | 20.32 |
| ES-122 | F | Sad | 25 | December | 2015 | 54.00 | 9.66 | 14.50 | 4.64 | 8.84 | 6.59 | 7.66 | 21.21 | 10.51 | 6.98 | 15.93 | 8.62 | 11.77 | 5.19 | 6.15 | 20.25 |
| ES-121 | F | — | 4 | April | 2016 | 47.90 | 9.47 | 13.95 | 4.44 | — | 6.29 | 7.25 | 20.31 | 10.03 | 6.53 | 14.84 | — | 10.85 | 4.93 | 5.79 | — |
| ES-120 | F | Ad | 18 | January | 2016 | 52.60 | 10.08 | 14.75 | 4.71 | 8.82 | 6.73 | 7.66 | 20.90 | 10.35 | 6.73 | 15.77 | 8.73 | — | 5.25 | — | — |
| ES-119 | M | Ad | 19 | July | 2016 | 51.00 | 9.33 | 13.36 | 4.59 | 8.35 | 6.27 | 7.14 | 19.68 | 9.62 | 6.63 | 14.78 | 8.23 | 10.68 | 5.10 | 5.80 | — |
| ES-118 | M | — | 1 | April | 2016 | 53.30 | 9.47 | 13.73 | — | 8.39 | 6.33 | 7.45 | 20.48 | 9.93 | 6.83 | 14.94 | 8.14 | 11.03 | 4.60 | — | 18.68 |
| ES-117 | F | — | 6 | April | 2016 | 53.30 | 9.15 | 14.03 | 4.37 | 8.44 | — | — | — | 10.15 | 6.75 | 15.47 | 8.44 | 11.55 | 5.13 | 5.93 | — |
| ES-115 | F | Sad | 23 | November | 2013 | 54.10 | 9.93 | — | 4.29 | 8.93 | 6.89 | 7.65 | 21.07 | 10.55 | 6.71 | — | 8.74 | — | — | — | 20.46 |
| ES-114 | F | — | 9 | February | 2014 | 52.10 | 9.18 | 14.07 | 4.38 | 8.64 | 6.38 | 7.84 | 21.01 | 10.12 | 6.76 | 15.85 | 9.03 | — | — | — | 20.26 |
| ES-113 | F | Ad | 5 | February | 2016 | 54.80 | 10.13 | 15.35 | 4.37 | 9.45 | — | — | — | 10.70 | 6.76 | 16.26 | 8.99 | — | — | — | — |
| ES-112 | F | Sad | 14 | February | 2016 | 54.80 | 9.35 | — | 4.29 | 8.89 | 6.56 | 7.72 | — | — | 6.84 | — | — | — | — | — | 19.94 |
| ES-111 | F | Ad | 14 | March | 2016 | 51.90 | 9.44 | 13.61 | 4.33 | 8.18 | 6.30 | 7.25 | 20.12 | 9.78 | 6.82 | 14.99 | 8.31 | 10.41 | 5.16 | 5.64 | 19.44 |
| ES-109 | F | — | 4 | January | 2016 | 55.20 | 9.36 | — | 4.33 | 8.67 | 6.32 | 7.72 | 20.99 | 10.47 | — | — | 8.79 | 11.72 | 4.86 | 5.61 | 20.30 |
| ES-108 | M | Ad | 8 | February | 2012 | 50.30 | 9.47 | 13.83 | 4.40 | 8.46 | 6.28 | 7.24 | 19.94 | 9.88 | 6.63 | 14.70 | 8.14 | 11.07 | 4.65 | 5.61 | 19.09 |
| ES-107 | M | Sad | 10 | February | 2005 | 53.30 | — | 13.74 | 4.47 | 8.43 | 6.23 | 7.36 | — | 10.56 | — | 15.40 | 8.08 | 11.25 | 5.26 | 6.04 | — |
| ES-106 | M | Sad | 12 | August | 2013 | 50.70 | — | 13.37 | — | — | 6.03 | 7.50 | — | 10.01 | 6.34 | 15.18 | 8.37 | 10.97 | 5.03 | 5.75 | 19.63 |
| ES-105 | M | Ad | 17 | February | 2013 | 52.70 | 9.26 | 14.25 | 4.21 | 8.29 | 6.44 | 7.58 | 20.87 | 9.93 | 7.07 | 15.53 | 8.44 | 10.87 | 5.13 | 5.80 | 19.70 |
| ES-104 | F | Ad | 25 | June | 2010 | 51.60 | 9.51 | 15.65 | 4.46 | 8.84 | 6.79 | 7.71 | — | 10.37 | — | 15.81 | 8.70 | 12.13 | 5.08 | 6.31 | 20.23 |
| ES-103 | M | Sad | 7 | August | 2012 | 53.00 | 9.61 | 13.83 | 4.37 | 8.37 | 6.24 | 7.45 | 20.67 | 10.19 | — | — | — | — | 4.97 | 5.52 | — |
| ES-102 | F | Sad | 10 | August | 2006 | 56.50 | 9.38 | — | 4.21 | 8.51 | — | 7.82 | — | 10.52 | — | 15.99 | 8.77 | — | — | — | 20.26 |
| ES-101 | M | Sad | 20 | December | 2012 | 51.00 | 9.33 | 13.99 | 4.42 | 8.59 | 6.51 | 7.44 | — | 9.49 | — | 15.02 | 8.29 | 11.39 | 4.91 | 6.03 | 19.32 |
| ES-100 | F | — | 14 | August | 2006 | 51.80 | — | — | 4.25 | 8.52 | 6.34 | 7.62 | — | 9.92 | — | 15.41 | 8.82 | 11.54 | 5.08 | 6.07 | — |

**Table S2.** Wing morphometric measurements of *C. serotinus* (n = 22). Linear measurements in cm, areas in cm². Abbreviations: Fa — forearm length; MC2–MC5 — metacarpal lengths; PP3–PP5 — proximal phalanx lengths; MP3 — medial phalanx of digit 3; DP3–DP5 — distal phalanx lengths; DG3–DG5 — total digit lengths; WL — wing length; HL — hand-wing length; AL — arm-wing length; WA — total wing area; HA — hand-wing area; AA — arm-wing area. Sex: F — female, M — male. Age: AD — adult, SD — subadult.

| **ID** | **Sex** | **Age** | **Fa** | **MC2** | **MC3** | **MC4** | **MC5** | **PP3** | **PP4** | **PP5** | **MP3** | **DP3** | **DP4** | **DP5** | **DG3** | **DG4** | **DG5** | **WL** | **HL** | **AL** | **WA** | **HA** | **AA** |
| --- | --- | --- | --- | --- | --- | --- | --- | --- | --- | --- | --- | --- | --- | --- | --- | --- | --- | --- | --- | --- | --- | --- | --- |
| SD_1 | F | AD | 5.121 | 4.325 | 5.048 | 4.760 | 4.565 | 1.873 | 1.556 | 1.243 | 1.519 | 1.000 | 1.291 | 0.938 | 8.770 | 7.466 | 6.239 | 12.955 | 8.608 | 4.364 | 62.572 | 31.985 | 31.043 |
| SD_5 | F | SD | 5.213 | 4.984 | 5.181 | 5.088 | 4.807 | 1.985 | 1.778 | 1.255 | 1.676 | 0.993 | 1.331 | 1.046 | 8.722 | 4.591 | 6.774 | 13.809 | 9.096 | 4.713 | 74.928 | 39.232 | 35.655 |
| SD_11 | F | AD | 5.170 | 5.075 | 5.082 | 4.961 | 4.568 | 2.005 | 1.808 | 1.347 | 1.606 | 1.104 | 1.277 | 1.035 | 9.251 | 7.881 | 6.722 | 13.725 | 9.117 | 4.608 | 72.444 | 36.730 | 35.814 |
| SD_21 | F | AD | 5.035 | 5.347 | 5.094 | 5.158 | 4.914 | 1.845 | 1.760 | 1.583 | 1.604 | 1.203 | 1.468 | 0.817 | 9.228 | 7.897 | 6.375 | 14.118 | 8.903 | 5.273 | 68.981 | 34.776 | 33.884 |
| SD_26 | F | AD | 5.144 | 4.707 | 4.967 | 4.738 | 4.346 | 1.854 | 1.801 | 1.358 | 1.523 | 0.970 | 1.165 | 0.880 | 9.182 | 7.701 | 6.510 | 13.767 | 9.007 | 4.777 | 65.587 | 33.098 | 32.380 |
| SD_30 | F | SD | 5.445 | 5.095 | 5.381 | 5.162 | 4.882 | 1.962 | 1.804 | 1.239 | 1.700 | 1.192 | 1.499 | 1.078 | 9.493 | 8.263 | 6.794 | 14.081 | 9.460 | 4.503 | 74.263 | 39.090 | 35.249 |
| SD_32 | F | AD | 4.982 | 4.788 | 4.836 | 4.719 | 4.380 | 1.697 | 1.541 | 1.069 | 1.429 | 1.101 | 1.305 | 1.032 | 8.654 | 7.427 | 6.189 | 13.078 | 8.648 | 4.446 | 65.864 | 33.741 | 32.347 |
| SD_37 | F | SD | 5.141 | 4.813 | 4.907 | 4.848 | 4.639 | 1.923 | 1.690 | 1.271 | 1.529 | 1.529 | 1.529 | 1.529 | 9.114 | 7.784 | 6.715 | 13.833 | 9.021 | 4.792 | 72.590 | 36.739 | 35.950 |
| SD_38 | F | AD | 5.257 | 5.053 | 5.157 | 5.137 | 4.796 | 1.852 | 1.668 | 1.308 | 1.591 | 1.104 | 1.271 | 0.920 | 9.339 | 7.948 | 6.588 | 13.901 | 9.142 | 4.759 | 71.281 | 35.225 | 35.826 |
| SD_41 | F | SD | 5.475 | 4.530 | 5.226 | 5.083 | 4.806 | 1.963 | 1.808 | 1.348 | 1.451 | 1.358 | 1.447 | 0.913 | 9.614 | 8.249 | 6.732 | 14.805 | 9.176 | 5.611 | 76.436 | 39.962 | 36.776 |
| SD_42 | F | SD | 5.204 | 4.103 | 5.028 | 4.856 | 4.653 | 1.845 | 1.603 | 1.067 | 1.424 | 0.999 | 1.073 | 0.685 | 8.949 | 7.404 | 6.190 | 13.624 | 8.357 | 5.195 | 77.748 | 37.376 | 40.332 |
| SD_2 | M | AD | 5.136 | 4.597 | 5.046 | 4.851 | 4.690 | 1.845 | 1.845 | 1.358 | 1.620 | 1.201 | 1.312 | 1.120 | 8.832 | 7.557 | 6.480 | 12.668 | 8.622 | 4.013 | 60.673 | 29.299 | 31.846 |
| SD_3 | M | AD | 5.106 | 4.370 | 4.995 | 4.802 | 4.364 | 1.758 | 1.662 | 1.248 | 1.761 | 0.779 | 1.259 | 0.985 | 8.892 | 7.568 | 6.090 | 13.304 | 8.722 | 4.591 | 63.739 | 33.074 | 30.918 |
| SD_8 | M | AD | 4.823 | 4.707 | 4.733 | 4.631 | 4.261 | 1.763 | 1.627 | 1.145 | 1.363 | 0.922 | 1.141 | 0.836 | 8.420 | 7.236 | 5.720 | 12.921 | 8.465 | 4.488 | 59.712 | 30.624 | 29.226 |
| SD_10 | M | AD | 5.037 | 4.775 | 4.981 | 4.901 | 4.539 | 1.956 | 1.663 | 1.226 | 1.415 | 1.103 | 1.309 | 0.748 | 9.181 | 7.745 | 6.258 | 13.564 | 8.861 | 4.670 | 64.442 | 33.939 | 30.476 |
| SD_16 | M | AD | 5.158 | 4.702 | 4.868 | 4.756 | 4.419 | 1.886 | 1.610 | 1.307 | 1.832 | 0.931 | 1.311 | 0.950 | 8.919 | 7.340 | 6.364 | 13.398 | 8.791 | 4.575 | 67.331 | 33.581 | 33.858 |
| SD_23 | M | SD | 4.749 | 4.450 | 4.720 | 4.461 | 4.227 | 1.715 | 1.557 | 1.110 | 1.370 | 0.723 | 1.157 | 0.733 | 8.454 | 7.185 | 5.952 | 12.804 | 8.425 | 4.401 | 55.735 | 27.464 | 28.266 |
| SD_25 | M | SD | 4.755 | 4.551 | 4.697 | 4.677 | 4.302 | 1.737 | 1.679 | 1.153 | 1.454 | 1.125 | 1.220 | 0.946 | 8.480 | 7.361 | 5.944 | 12.775 | 8.473 | 4.350 | 60.156 | 29.055 | 31.375 |
| SD_34 | M | AD | 4.964 | 4.690 | 4.933 | 4.838 | 4.448 | 1.683 | 1.360 | 1.096 | 1.428 | 1.201 | 1.321 | 0.857 | 8.925 | 7.436 | 6.100 | 13.373 | 8.904 | 4.469 | 62.307 | 29.920 | 32.626 |
| SD_35 | M | AD | 5.052 | 4.579 | 4.818 | 4.618 | 4.395 | 1.827 | 1.549 | 1.251 | 1.527 | 1.131 | 1.314 | 0.946 | 8.921 | 7.336 | 6.189 | 13.204 | 8.954 | 4.311 | 60.465 | 34.110 | 26.484 |
| SD_44 | M | SD | 5.077 | 4.306 | 4.951 | 4.945 | 4.531 | 1.924 | 1.607 | 1.263 | 1.352 | 1.124 | 1.292 | 0.974 | 8.945 | 7.655 | 6.384 | 13.569 | 8.897 | 4.672 | 62.118 | 31.751 | 30.093 |
| SD_45 | M | SD | 5.121 | 4.793 | 5.025 | 4.895 | 4.575 | 1.833 | 1.663 | 1.274 | 1.618 | 0.873 | 1.281 | 0.898 | 8.928 | 7.652 | 6.444 | 13.555 | 8.937 | 4.585 | 68.956 | 33.590 | 35.394 |

**Table S3.** Results of two-way ANOVA (Sex × Age) for craniometric parameters of *C. serotinus*. Only specimens with known age were included (n = 97). F — F-statistic; p — p-value; η² — partial eta-squared (effect size); Int — Sex × Age interaction. Significance: * p < 0.05, ** p < 0.01, *** p < 0.001.

| **Parameter** | **n** | **F(Sex)** | **p(Sex)** | **η²(Sex)** | **F(Age)** | **p(Age)** | **η²(Age)** | **F(Int)** | **p(Int)** | **η²(Int)** |
| --- | --- | --- | --- | --- | --- | --- | --- | --- | --- | --- |
| Ra | 92 | 13.98 | **<0.001***** | 0.137 | 0.25 | 0.616 | 0.003 | 0.75 | 0.389 | 0.008 |
| Brain | 93 | 1.05 | 0.308 | 0.012 | 2.03 | 0.157 | 0.022 | 0.68 | 0.411 | 0.008 |
| Z-Z | 86 | 7.83 | **0.006**** | 0.087 | 5.69 | **0.019*** | 0.065 | 1.28 | 0.262 | 0.015 |
| B-B | 96 | 5.25 | **0.024*** | 0.054 | 4.09 | **0.046*** | 0.043 | 0.77 | 0.382 | 0.008 |
| Mg-Mg | 93 | 13.07 | **<0.001***** | 0.128 | 3.46 | 0.066 | 0.037 | 0.66 | 0.419 | 0.007 |
| Cs-Cs | 92 | 9.57 | **0.003**** | 0.098 | 2.14 | 0.147 | 0.024 | 1.69 | 0.196 | 0.019 |
| Cs-Mg | 94 | 8.79 | **0.004**** | 0.089 | 2.45 | 0.121 | 0.026 | 0.42 | 0.520 | 0.005 |
| Pr-La | 75 | 1.88 | 0.175 | 0.026 | 2.65 | 0.108 | 0.036 | 1.56 | 0.215 | 0.022 |
| A-Sq | 96 | 6.76 | **0.011*** | 0.068 | 1.41 | 0.237 | 0.015 | 6.50 | **0.012*** | 0.066 |
| Shpb | 80 | 1.01 | 0.318 | 0.013 | 8.81 | **0.004**** | 0.104 | 1.70 | 0.196 | 0.022 |
| In-Cn | 88 | 14.64 | **<0.001***** | 0.148 | 5.21 | **0.025*** | 0.058 | 3.08 | 0.083 | 0.035 |
| Ci-Mi | 93 | 10.20 | **0.002**** | 0.103 | 3.26 | 0.074 | 0.035 | 0.28 | 0.599 | 0.003 |
| Ci-Co | 74 | 4.95 | **0.029*** | 0.066 | 4.75 | **0.033*** | 0.064 | 0.74 | 0.393 | 0.010 |
| Co-Cn | 76 | 11.16 | **0.001**** | 0.134 | 4.02 | **0.049*** | 0.053 | 0.09 | 0.770 | 0.001 |
| Co-R | 73 | 10.11 | **0.002**** | 0.128 | 0.29 | 0.589 | 0.004 | 0.42 | 0.518 | 0.006 |
| Pr-Fm | 87 | 4.36 | **0.040*** | 0.050 | 4.81 | **0.031*** | 0.055 | 4.18 | **0.044*** | 0.048 |

**Table S4.** Results of two-way ANOVA (Sex × Age) for wing morphometric parameters of *C. serotinus* (n = 22). F — F-statistic; p — p-value; η² — partial eta-squared (effect size); Int — Sex × Age interaction. Significance: * p < 0.05, ** p < 0.01, *** p < 0.001.

| **Parameter** | **n** | **F(Sex)** | **p(Sex)** | **η²(Sex)** | **F(Age)** | **p(Age)** | **η²(Age)** | **F(Int)** | **p(Int)** | **η²(Int)** |
| --- | --- | --- | --- | --- | --- | --- | --- | --- | --- | --- |
| Fa | 22 | 11.62 | **0.003**** | 0.392 | 0.85 | 0.368 | 0.045 | 5.38 | **0.032*** | 0.230 |
| MC2 | 22 | 2.89 | 0.107 | 0.138 | 0.95 | 0.342 | 0.050 | 0.42 | 0.524 | 0.023 |
| MC3 | 22 | 10.62 | **0.004**** | 0.371 | 0.60 | 0.450 | 0.032 | 1.71 | 0.207 | 0.087 |
| MC4 | 22 | 7.34 | **0.014*** | 0.290 | 0.56 | 0.465 | 0.030 | 0.38 | 0.544 | 0.021 |
| MC5 | 22 | 11.18 | **0.004**** | 0.383 | 1.50 | 0.237 | 0.077 | 1.23 | 0.281 | 0.064 |
| PP3 | 22 | 4.59 | **0.046*** | 0.203 | 1.24 | 0.280 | 0.065 | 1.19 | 0.289 | 0.062 |
| PP4 | 22 | 3.38 | 0.082 | 0.158 | 0.57 | 0.461 | 0.031 | -0.08 | 1.000 | -0.005 |
| PP5 | 22 | 1.41 | 0.251 | 0.072 | 1.04 | 0.321 | 0.055 | 0.47 | 0.500 | 0.026 |
| MP3 | 22 | 0.24 | 0.629 | 0.013 | 0.64 | 0.433 | 0.035 | 1.22 | 0.283 | 0.064 |
| DP3 | 22 | 3.22 | 0.089 | 0.152 | 0.35 | 0.560 | 0.019 | 1.83 | 0.192 | 0.092 |
| DP4 | 22 | 2.01 | 0.173 | 0.100 | 0.30 | 0.591 | 0.016 | 1.49 | 0.238 | 0.077 |
| DP5 | 22 | 1.17 | 0.293 | 0.061 | 0.44 | 0.514 | 0.024 | 0.81 | 0.380 | 0.043 |
| DG3 | 22 | 6.42 | **0.021*** | 0.263 | 0.00 | 0.975 | 0.000 | 1.25 | 0.278 | 0.065 |
| DG4 | 22 | 0.02 | 0.879 | 0.001 | 0.51 | 0.485 | 0.027 | 0.54 | 0.473 | 0.029 |
| DG5 | 22 | 11.91 | **0.003**** | 0.398 | 1.85 | 0.190 | 0.093 | 0.10 | 0.758 | 0.005 |
| WL | 22 | 11.30 | **0.003**** | 0.386 | 2.20 | 0.155 | 0.109 | 0.86 | 0.367 | 0.045 |
| HL | 22 | 3.80 | 0.067 | 0.174 | 0.15 | 0.708 | 0.008 | 0.57 | 0.459 | 0.031 |
| AL | 22 | 7.53 | **0.013*** | 0.295 | 2.14 | 0.160 | 0.106 | -0.09 | 1.000 | -0.005 |
| WA | 22 | 34.82 | **<0.001***** | 0.659 | 7.57 | **0.013*** | 0.296 | 4.77 | **0.042*** | 0.209 |
| HA | 22 | 31.40 | **<0.001***** | 0.636 | 4.65 | **0.045*** | 0.205 | 9.71 | **0.006**** | 0.350 |
| AA | 22 | 16.53 | **<0.001***** | 0.479 | 5.07 | **0.037*** | 0.220 | 0.28 | 0.606 | 0.015 |

**Table S5.** Sensitivity analysis of sexual dimorphism in cranial and forearm dimensions of *Cnephaeus serotinus* under three sampling scenarios. Comparison of sexual dimorphism estimates between (1) the full sample, (2) the sample excluding August subadults and August individuals of unknown age (i.e., specimens most likely to be this-year-born with incomplete cranial ossification), and (3) confirmed adults only. Welch’s t-test was used for between-sex comparisons; p-values were adjusted for multiple testing using the Benjamini–Hochberg FDR procedure. Cohen’s *d* > 0 indicates female-biased dimorphism. Significant comparisons after FDR correction (p < 0.05) are marked with an asterisk and shown in bold.

| **Variable** | **(1) Full sample (n = 137; 70 F, 67 M)** | | | | | **(2) Excl. August Sad + Unknown (n = 112; 58 F, 54 M)** | | | | | **(3) Confirmed adults only (n = 39; 20 F, 19 M)** | | | | |
| --- | --- | --- | --- | --- | --- | --- | --- | --- | --- | --- | --- | --- | --- | --- | --- |
|  | **F mean ± SD** | **M mean ± SD** | **Diff (%)** | **Cohen's d** | **p (FDR)** | **F mean ± SD** | **M mean ± SD** | **Diff (%)** | **Cohen's d** | **p (FDR)** | **F mean ± SD** | **M mean ± SD** | **Diff (%)** | **Cohen's d** | **p (FDR)** |
| Ra | **53.12 ± 1.68** | **52.00 ± 1.43** | **+2.14** | **+0.710** | **0.0008*** | **53.09 ± 1.71** | **52.03 ± 1.47** | **+2.04** | **+0.666** | **0.0036*** | **53.47 ± 1.29** | **51.97 ± 1.09** | **+2.89** | **+1.257** | **0.0027*** |
| Brain | 9.55 ± 0.25 | 9.49 ± 0.22 | +0.60 | +0.242 | 0.1870 | 9.56 ± 0.26 | 9.51 ± 0.22 | +0.48 | +0.187 | 0.3345 | 9.64 ± 0.27 | 9.54 ± 0.20 | +1.00 | +0.396 | 0.2444 |
| Z-Z | **14.25 ± 0.47** | **14.04 ± 0.52** | **+1.46** | **+0.414** | **0.0356*** | 14.28 ± 0.46 | 14.11 ± 0.53 | +1.17 | +0.333 | 0.1136 | **14.48 ± 0.53** | **14.09 ± 0.35** | **+2.77** | **+0.863** | **0.0391*** |
| B-B | 4.40 ± 0.14 | 4.43 ± 0.19 | -0.80 | -0.217 | 0.2160 | 4.40 ± 0.13 | 4.46 ± 0.18 | -1.25 | -0.349 | 0.0901 | 4.41 ± 0.13 | 4.52 ± 0.21 | -2.47 | -0.647 | 0.0798 |
| Mg-Mg | **8.65 ± 0.25** | **8.52 ± 0.21** | **+1.55** | **+0.577** | **0.0022*** | **8.68 ± 0.25** | **8.54 ± 0.21** | **+1.61** | **+0.595** | **0.0061*** | 8.75 ± 0.29 | 8.56 ± 0.24 | +2.19 | +0.705 | 0.0652 |
| Cs-Cs | **6.53 ± 0.19** | **6.41 ± 0.21** | **+1.85** | **+0.593** | **0.0021*** | **6.54 ± 0.18** | **6.45 ± 0.20** | **+1.54** | **+0.512** | **0.0151*** | 6.56 ± 0.16 | 6.48 ± 0.21 | +1.14 | +0.397 | 0.2444 |
| Cs-Mg | **7.60 ± 0.20** | **7.48 ± 0.16** | **+1.54** | **+0.639** | **0.0010*** | **7.62 ± 0.19** | **7.49 ± 0.18** | **+1.67** | **+0.686** | **0.0023*** | 7.63 ± 0.18 | 7.52 ± 0.19 | +1.35 | +0.545 | 0.1306 |
| Pr-La | **20.86 ± 0.51** | **20.65 ± 0.43** | **+1.01** | **+0.447** | **0.0382*** | 20.87 ± 0.50 | 20.67 ± 0.45 | +0.96 | +0.416 | 0.0757 | 21.00 ± 0.45 | 20.71 ± 0.39 | +1.43 | +0.709 | 0.0798 |
| A-Sq | **10.33 ± 0.29** | **10.16 ± 0.26** | **+1.64** | **+0.611** | **0.0013*** | **10.34 ± 0.29** | **10.18 ± 0.27** | **+1.58** | **+0.568** | **0.0067*** | **10.45 ± 0.23** | **10.13 ± 0.20** | **+3.18** | **+1.480** | **0.0007*** |
| Shpb | 6.78 ± 0.21 | 6.69 ± 0.25 | +1.28 | +0.370 | 0.0666 | 6.79 ± 0.20 | 6.71 ± 0.25 | +1.10 | +0.331 | 0.1211 | 6.83 ± 0.17 | 6.82 ± 0.20 | +0.09 | +0.033 | 0.9227 |
| In-Cn | **15.64 ± 0.38** | **15.31 ± 0.34** | **+2.16** | **+0.922** | **0.0000*** | **15.68 ± 0.36** | **15.36 ± 0.31** | **+2.10** | **+0.948** | **0.0001*** | **15.81 ± 0.31** | **15.40 ± 0.31** | **+2.63** | **+1.296** | **0.0027*** |
| Ci-Mi | **8.60 ± 0.21** | **8.44 ± 0.20** | **+1.88** | **+0.784** | **0.0001*** | **8.61 ± 0.20** | **8.46 ± 0.21** | **+1.83** | **+0.759** | **0.0013*** | 8.65 ± 0.19 | 8.50 ± 0.22 | +1.74 | +0.719 | 0.0652 |
| Ci-Co | **11.57 ± 0.47** | **11.35 ± 0.32** | **+1.97** | **+0.564** | **0.0080*** | **11.60 ± 0.42** | **11.37 ± 0.34** | **+1.96** | **+0.586** | **0.0132*** | 11.75 ± 0.51 | 11.43 ± 0.43 | +2.74 | +0.662 | 0.0991 |
| Co-Cn | **5.15 ± 0.21** | **4.98 ± 0.17** | **+3.40** | **+0.891** | **0.0001*** | **5.16 ± 0.21** | **5.01 ± 0.16** | **+3.04** | **+0.799** | **0.0019*** | **5.21 ± 0.21** | **5.03 ± 0.17** | **+3.64** | **+0.969** | **0.0303*** |
| Co-R | **6.05 ± 0.28** | **5.86 ± 0.18** | **+3.36** | **+0.856** | **0.0002*** | **6.05 ± 0.27** | **5.88 ± 0.15** | **+2.79** | **+0.756** | **0.0035*** | **6.09 ± 0.29** | **5.86 ± 0.17** | **+3.87** | **+0.940** | **0.0391*** |
| Pr-Fm | **20.09 ± 0.46** | **19.84 ± 0.40** | **+1.27** | **+0.583** | **0.0040*** | **20.11 ± 0.46** | **19.86 ± 0.42** | **+1.27** | **+0.573** | **0.0100*** | **20.29 ± 0.39** | **19.90 ± 0.32** | **+1.92** | **+1.063** | **0.0154*** |
| **N significant (FDR < 0.05)** | **13 / 16** | | | | | **11 / 16** | | | | | **7 / 16** | | | | |

***Note:*** *F, females; M, males; SD, standard deviation; Diff (%), percentage difference of female mean relative to male mean; Cohen’s d, effect size of sex difference (positive = female-biased); FDR, Benjamini–Hochberg adjusted p-value. Bold cells indicate sex differences significant at FDR < 0.05.*

**Table S6.** Comparison of craniometric and forearm dimensions between August-collected immature individuals of *C. serotinus* and conspecifics of comparable age collected in other months. Three independent comparisons were performed: (1) August subadults vs. subadults from other months; (2) August individuals of unknown age vs. unknown-age individuals from other months; (3) August immature + unknown-age individuals (pooled) vs. corresponding pooled subset from other months. Between-group comparisons used Welch’s t-test (parametric) and Mann–Whitney U test (non-parametric); effect sizes are reported as Cohen’s *d*, where *d* < 0 indicates that August individuals are smaller. Significant differences (p < 0.05) are marked with an asterisk and shown in bold.

| **Variable** | **(1) Aug Sad (n = 8) vs. Other Sad (n = 50)** | | | | | | **(2) Aug Unknown (n = 17) vs. Other Unknown (n = 23)** | | | | | | **(3) Aug pooled young+unk (n = 25) vs. Other pooled (n = 73)** | | | | | |
| --- | --- | --- | --- | --- | --- | --- | --- | --- | --- | --- | --- | --- | --- | --- | --- | --- | --- | --- |
|  | **Aug mean ± SD** | **Other mean ± SD** | **Diff (%)** | **Cohen's d** | **t-test p** | **MW U p** | **Aug mean ± SD** | **Other mean ± SD** | **Diff (%)** | **Cohen's d** | **t-test p** | **MW U p** | **Aug mean ± SD** | **Other mean ± SD** | **Diff (%)** | **Cohen's d** | **t-test p** | **MW U p** |
| Ra | 53.31 ± 1.59 | 52.46 ± 1.75 | +1.63 | +0.494 | 0.1959 | 0.2515 | 51.94 ± 1.34 | 52.50 ± 2.13 | -1.06 | -0.291 | 0.4740 | 0.2625 | 52.67 ± 1.59 | 52.47 ± 1.82 | +0.39 | +0.116 | 0.6668 | 0.9740 |
| Brain | 9.49 ± 0.14 | 9.53 ± 0.23 | -0.42 | -0.180 | 0.5437 | 0.7331 | 9.46 ± 0.22 | 9.44 ± 0.25 | +0.17 | +0.069 | 0.8459 | 0.6667 | 9.47 ± 0.19 | 9.50 ± 0.24 | -0.35 | -0.147 | 0.5247 | 0.3885 |
| Z-Z | 13.71 ± 0.45 | 14.12 ± 0.40 | -2.87 | -0.996 | 0.0774 | 0.0536 | 13.89 ± 0.35 | 14.20 ± 0.69 | -2.18 | -0.520 | 0.1086 | 0.1543 | **13.83 ± 0.38** | **14.14 ± 0.50** | **-2.23** | **-0.652** | **0.0083*** | **0.0126*** |
| B-B | 4.34 ± 0.10 | 4.41 ± 0.15 | -1.42 | -0.427 | 0.1951 | 0.5112 | 4.35 ± 0.19 | 4.43 ± 0.15 | -1.80 | -0.480 | 0.1611 | 0.1564 | 4.35 ± 0.17 | 4.41 ± 0.15 | -1.47 | -0.424 | 0.0985 | 0.0873 |
| Mg-Mg | 8.56 ± 0.25 | 8.56 ± 0.22 | -0.11 | -0.040 | 0.9304 | 0.8399 | **8.46 ± 0.18** | **8.62 ± 0.19** | **-1.86** | **-0.878** | **0.0103*** | **0.0136*** | 8.49 ± 0.20 | 8.58 ± 0.21 | -1.09 | -0.447 | 0.0589 | 0.0783 |
| Cs-Cs | **6.27 ± 0.24** | **6.49 ± 0.21** | **-3.39** | **-1.032** | **0.0542** | **0.0285*** | 6.39 ± 0.20 | 6.47 ± 0.20 | -1.18 | -0.383 | 0.2615 | 0.2027 | **6.36 ± 0.21** | **6.48 ± 0.21** | **-1.96** | **-0.611** | **0.0181*** | **0.0133*** |
| Cs-Mg | 7.49 ± 0.16 | 7.52 ± 0.18 | -0.32 | -0.136 | 0.6980 | 0.5501 | **7.46 ± 0.17** | **7.61 ± 0.21** | **-1.90** | **-0.763** | **0.0236*** | **0.0190*** | **7.47 ± 0.16** | **7.54 ± 0.19** | **-0.96** | **-0.390** | **0.0773** | **0.0500*** |
| Pr-La | 20.64 ± 0.28 | 20.68 ± 0.51 | -0.17 | -0.071 | 0.8355 | 0.7971 | 20.61 ± 0.50 | 20.78 ± 0.50 | -0.85 | -0.352 | 0.4319 | 0.1984 | 20.62 ± 0.43 | 20.71 ± 0.50 | -0.44 | -0.184 | 0.5303 | 0.3475 |
| A-Sq | 10.26 ± 0.18 | 10.22 ± 0.33 | +0.35 | +0.115 | 0.6617 | 0.7046 | 10.12 ± 0.24 | 10.28 ± 0.26 | -1.55 | -0.632 | 0.0623 | 0.0707 | 10.17 ± 0.23 | 10.24 ± 0.31 | -0.70 | -0.249 | 0.2334 | 0.2736 |
| Shpb | 6.47 ± 0.16 | 6.70 ± 0.24 | -3.39 | -0.976 | 0.0523 | 0.0581 | 6.69 ± 0.31 | 6.73 ± 0.25 | -0.55 | -0.139 | 0.7723 | 0.8525 | 6.62 ± 0.28 | 6.71 ± 0.24 | -1.38 | -0.375 | 0.3098 | 0.2699 |
| In-Cn | 15.35 ± 0.38 | 15.46 ± 0.35 | -0.70 | -0.307 | 0.5022 | 0.4037 | 15.26 ± 0.45 | 15.53 ± 0.42 | -1.74 | -0.619 | 0.0739 | 0.0553 | **15.29 ± 0.43** | **15.48 ± 0.37** | **-1.25** | **-0.500** | **0.0615** | **0.0416*** |
| Ci-Mi | 8.49 ± 0.20 | 8.50 ± 0.21 | -0.14 | -0.057 | 0.8867 | 0.8103 | 8.47 ± 0.22 | 8.54 ± 0.23 | -0.83 | -0.311 | 0.3520 | 0.2438 | 8.47 ± 0.21 | 8.51 ± 0.22 | -0.44 | -0.175 | 0.4652 | 0.3599 |
| Ci-Co | 11.31 ± 0.62 | 11.40 ± 0.29 | -0.78 | -0.256 | 0.7407 | 0.4200 | 11.31 ± 0.40 | 11.45 ± 0.36 | -1.18 | -0.361 | 0.3220 | 0.2020 | 11.31 ± 0.46 | 11.42 ± 0.31 | -0.91 | -0.292 | 0.3538 | 0.1208 |
| Co-Cn | 5.01 ± 0.10 | 5.04 ± 0.20 | -0.70 | -0.182 | 0.5038 | 0.5964 | **4.93 ± 0.22** | **5.08 ± 0.19** | **-2.95** | **-0.731** | **0.0469*** | **0.0519** | **4.96 ± 0.19** | **5.06 ± 0.20** | **-1.98** | **-0.507** | **0.0475*** | **0.0394*** |
| Co-R | 5.91 ± 0.37 | 5.95 ± 0.23 | -0.67 | -0.156 | 0.7901 | 0.7670 | 5.90 ± 0.30 | 5.96 ± 0.18 | -0.94 | -0.233 | 0.5295 | 0.3111 | 5.91 ± 0.31 | 5.95 ± 0.21 | -0.80 | -0.194 | 0.5176 | 0.3746 |
| Pr-Fm | 19.89 ± 0.29 | 19.90 ± 0.43 | -0.06 | -0.029 | 0.9292 | 0.8299 | 19.81 ± 0.43 | 20.00 ± 0.60 | -0.96 | -0.347 | 0.3623 | 0.2052 | 19.84 ± 0.37 | 19.93 ± 0.48 | -0.44 | -0.190 | 0.4487 | 0.3481 |
| **N variables with p < 0.05 (t-test)** | **0 / 16** | | | | | | **3 / 16** | | | | | | **3 / 16** | | | | | |

***Note:*** *Sad, subadults (this-year-born individuals with completed postnatal growth); Unknown, individuals for which age could not be reliably determined; SD, standard deviation; Diff (%), percentage difference of August-group mean relative to non-August-group mean (negative values indicate that August individuals are smaller); Cohen’s d, effect size; MW U, Mann–Whitney U test. Tests were performed without multiple-testing correction within this exploratory analysis. Bold and asterisked p-values indicate significance at α = 0.05.*

**Note:** Partial η² interpretation according to Cohen (1988): small effect (0.01–0.06), medium effect (0.06–0.14), large effect (>0.14). For skulls, Age: Ad — adult, Sad — subadult. For wings, Age: AD — adult, SD — subadult.
